# Dataset on geosynthetic material debris contamination of the South-East Baltic shore

**DOI:** 10.1016/j.dib.2021.107778

**Published:** 2022-01-01

**Authors:** Boris Chubarenko, Alexander Kileso, Elena Esiukova, Vasiliy Pinchuk, Franz-Georg Simon

**Affiliations:** a Shirshov Institute of Oceanology, Russian Academy of Sciences, Moscow 117997, Russia; b Immanuel Kant Baltic Federal University, Kaliningrad 236041, Russia; c BAM Federal Institute for Materials Research and Testing, Berlin 12200, Germany

**Keywords:** Geosynthetics, Geotextiles, Sandy beach, Contamination, Marine littering

## Abstract

The database gives information on the contamination of the shore of the South-Eastern Baltic with the debris of geosynthetic materials for the period 2018–2020. This new type of coastal pollution enters the natural environment due to the destruction of coastal protection structures and construction activities. The database contains sections: (1) a list of types of geosynthetic material residues, their photographic images and photographs illustrating examples of finds in natural conditions [1 List_geosynthetic_debris_SEB], (2) monitoring data on the contamination of the beach strip with the debris of geotextiles, braids from gabions, geocontainers (big bags), geocells and geogrids for the beaches of the South-Eastern Baltic for the period 2018–2020 [2 Monitoring_geosynthetic_debris_SEB]; (3) statistical distributions of the found geosynthetic debris by size [3 Scales_geosynthetic_debris_SEB] and (4) results of test surveys on the shores of Lithuania and Poland adjacent to Kaliningrad Oblast. All data refer to the beaches of the Kaliningrad Oblast (Russia), including the Russian parts of the Vistula and Curonian Spits, but also contains information on a one-time assessment of the pollution of the beaches of the adjacent territories: the Polish shore from the Poland-Russia border on the Vistula Spit to the mouth of the Vistula River, the Lithuanian shore from the border Lithuania-Russia on the Curonian Spit to the border of Latvia-Lithuania. Materials were collected during field surveys within the ERANET-RUS_Plus joint project EI-GEO, ID 212 (RFBR 18-55-76002 ERA_a, BMBF 01DJ18005).

**Specifications Table**

**Table:** 

Subject	Environmental Science, Ecology, Earth Science
Specific subject area	Geosynthetic material debris contamination of the marine environment
Type of data	Table Image Graph
How data were acquired	Field data collection: samples of the geosynthetic debris were collected at the beaches by a group of observers, transported to the laboratory and classified.
Data format	Raw data.
Parameters for data collection	Samples collection was made on the beaches in the summer months in 2018, 2019, 2020. The shore is non-tidal. The weather was calm, with no wind-wave swash.
Description of data collection	Fragments of geosynthetic materials (not smaller than 1 cm) were collected during continuous visual scanning assumed a continuous passage along the entire coastline by a group of three observers. For each detected geosynthetic sample, the following parameters were recorded: the type of geosynthetic sample, geometrical dimensions (length and area), number of the coastline subsection where this sample was found, position on the beach (in % of the distance from the waterline, 100% is at the beach back). The photograph was taken, and the sample was collected for further laboratory analysis.
Data source location	Institution: Shirshov Institute of Oceanology, Russian Academy of Sciences City/Town/Region: Kaliningrad Country: Russian Federation Latitude and longitude (and GPS coordinates, if possible) for collected samples/data: The rectangular covered the study area (sandy beaches at the non-tidal shore of the Kaliningrad Oblast, Russia, in the South-Eastern Baltic) is described by coordinates of the left down corner (N 54.490266, E 19.690178) and the right top corner (N 55.253276, E 20.925951).
Data accessibility	Repository name: Mendeley Data identification number: DOI: 10.17632/bxzt2fr4hg.1 Direct URL to data: http://dx.doi.org/10.17632/bxzt2fr4hg.1
Related research article	E. Esiukova, B. Chubarenko, F.-G. Simon, Debris of geosynthetic materials on the shore of South-Eastern Baltic (Kaliningrad Oblast, Russian Federation). [In] Proc. of 7th IEEE/OES Baltic Symposium ``Clean and Safe Baltic Sea and Energy Security for the Baltic countries''. 12–15 June 2018, Klaipėda, Lithuania. IEEE Xplore Digital Library (2018) 1–6. 10.1109/BALTIC.2018.8634842 P. Scholz, I. Putna-Nimane, I. Barda, I. Liepina-Leimane, E. Strode, A. Kileso, E. Esiukova, B. Chubarenko, I. Purina, F.-G. Simon. Materials 14 (3) (2021) 634, doi:10.3390/ma14030634

**Value of the Data**

• The data are useful for policymakers to develop beach cleanup programs and programs to prevent possible coastal zone contamination. The data provided describe the level of contamination with the debris of geosynthetic materials on the beaches of the South-Eastern Baltic region (Kaliningrad Oblast, Russia and Polish and Lithuanian coasts adjacent to Kaliningrad Oblast), including the Curonian Spit UNESCO National Park. This new type of coastal contamination enters the natural environment due to the destruction of coastal protection structures and construction activities. The rate of contamination in surface beach sands is documented.
• The data are useful for researchers comparing beach contamination status along the Baltic Sea (or European seas). Data are given for summer periods of 2018–2020 and show the interannual variability of contamination level.
• The data are helpful for researchers to understand the general scheme of the transport by sea currents in the South-Eastern Baltic region. It can be used for hydrodynamic model calibration or validation.

## 1 Data Description

The database contains sections, which are presented in four separate files.

The first file [1 List_geosynthetic_debris_SEB] contains information about types of geosynthetic material debris found on the shore of the Kaliningrad Oblast (Russia, South-East Baltic) during field surveys in the 2018–2020 ERANET-RUS_Plus joint project EI-GEO, ID 212 (RFBR 18-55-76002 ERA_a, BMBF 01DJ18005). Photographic images of different types of geosynthetics and photographs illustrating examples of finds in natural conditions are included. Examples of the geotextile material debris found on the shore of the Kaliningrad Oblast are illustrated on Figs. 1.1–1.7. Examples of the gabion plastic coating fragments are presented on Figs. 1.8–1.10. Different types of woven geocontainers are presented on Figs. 1.11–1.13. Examples of geocells debris (Figs. 1.14–1.16) and geomats debris (Fig. 1.17) are presented. All figures mentioned in this paragraph are in the file.

The second folder [2 Monitoring_geosynthetic_debris_SEB] describes the data on the contamination of the beach strip with geosynthetic debris for the period 2018–2020. These are remnants of geotextiles, braids from gabions, geocontainers (big bags), geocells and geogrids found on the beaches of the South-Eastern Baltic in 2018–2020. The database in the form of the MS Excel workbook [2 Monitoring_geosynthetic_debris_SEB.xlsx] has the following structure.

Three MS Excel lists (``2018'', ``2019'', ``2020'') contain information about numbers of samples of geosynthetic material debris found on the shore of the Kaliningrad Region (Russia, South-East Baltic) during field surveys in 2018–2020.

**Fig. 1:**
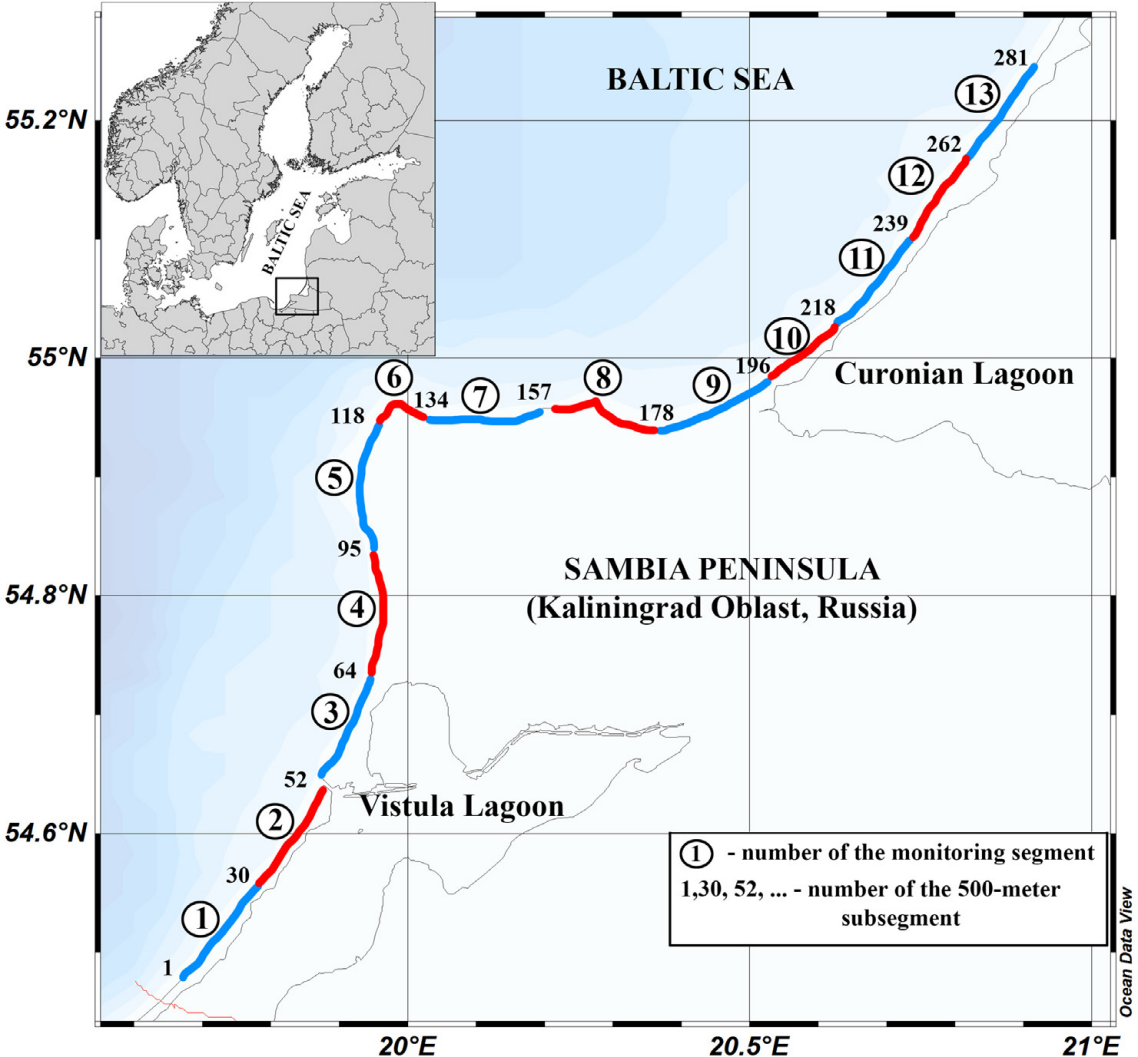
Monitoring field design in the South-Eastern Baltic. The numbers of the monitoring shore segments are in circles. The numbers of reference points mark the end of the monitoring segment.
‐ Coordinates (WGS 84) of the center of the subsegment (columns ``Latitude [degree]'', ``Longitude [degree]'')
‐ Number of samples of geosynthetic material of different types (Geotextile, Gabion coating, Geocontainer, Geocell, Geomat) found on corresponding subsegment (columns ``Geotextile [numbers]'', ``Gabion coating [numbers]'', ``Geocontainer [numbers]'', ``Geocell [numbers]'', ``Geomat [numbers]'').

Each MS Excel list has information about:

‐ The number of the 500 m coastline subsegment (column ``№ Subsegment''). The approximate position of the subsegments is shown in Fig. 1, which presents the segments monitored during one day. The numbers of the reference point marked the northern end of coastline subsegment (this number is also the number of coasline subsegment) are also indicated.

These data are also provided in «Comma-Separated Values» (CSV) format for each year separately. Data for 2018 - [2a Monitoring_geosynthetic_debris_SEB_2018.csv], data for 2019 - [2a Monitoring_geosynthetic_debris_SEB_2019.csv] and data for 2020 - [2a Monitoring_geosynthetic_debris_SEB_2020.csv].

The third file [3 Scales_geosynthetic_debris_SEB] demonstrates the statistical distributions of the found geosynthetic debris by spatial size. For geotextile (Fig. 3.1) and geo-container (Fig. 3.2), variations of a sample area (cm^2^) are presented, while for gabions (Fig. 3.3), the variations of a sample length (cm) are presented. The statistics on sample size are presented in the form of a box-and-whisker diagram for debris of geotextile, geo-container and gabion plastic coating for each monitoring year. On each box-and-whisker diagram, the label inside the box indicates the value of the median sample size. Upper and lower whiskers correspond to the maximum and minimum values. The upper whiskers are also labelled. The diagrams were not prepared for other types of geosynthetic materials (geocells, geo-mats) due to the small number of collected samples. The samples usually have such a complicated geometry. The dimensions of the samples were estimated and rounded. All figures mentioned in this paragraph are in the file.

The fourth file [4 Monitoring Poland and Lithuania] contains information about the types of geosynthetic material debris found on the Polish and Lithuanian coasts adjacent to Kaliningrad Oblast (South-East Baltic) during field surveys in May-June 2019.

The position of the twelve test 1-km segments at the Lithuanian part of the shore of the Southeastern Baltic and seven test segments of various lengths at the Polish part of the neighbouring shore are illustrated on (Fig. 4.1). Information about test field surveys (coordinates, length of monitoring segments and time of the surveys) on the shore of the Lithuanian and Polish coasts are presented in (Table 4.1). The number of geosynthetic material debris of the various types which was found on the test segments on Lithuanian and Polish coasts are presented in (Table 4.2). All tables and figures mentioned in this paragraph is in the fourth file mentioned above.

Elena Esiukova took all photos.

## 2 Experimental Design, Materials and Methods

The study area (Fig. 1) in the South-Eastern Baltic included the beaches of the Kaliningrad Oblast (Russia) and also contains the beach segments on the adjacent territories: the Polish shore from the Poland-Russia border on the Vistula Spit to the mouth of the Vistula River, the Lithuanian shore from the border Lithuania-Russia on the Curonian Spit to the border of Latvia-Lithuania. Therefore, the transboundary shores of the sandy barrier, the Vistula and Curonian spits were studied as a whole.

The main activity was applied to the shore of the Kaliningrad Oblast, which was divided into 13 monitoring segments of nearly equal length. The length of such a specific monitoring segment was approximately 10 km (± 1.5 km), making it possible to efficiently carry out work on it in one expedition day. The average time spent on one monitoring segment is about 6-8 hours. In addition, the monitoring segments were assigned to reach the starting and ending points of the section by road.

The segments were numbered in the direction from south to north (from west to east), starting from the state border with the Republic of Poland (on the Vistula Spit) and ending at the state border with the Republic of Lithuania (on the Curonian Spit).

There are two sections (No. 1-2) on the Russian side of the Vistula Spit; 3 sections on the western shore of the Sambia Peninsula (No. 3-5); 4 sections on the northern shore (No. 6-9) of the Sambia Peninsula; 4 sections (No. 10-13) on the Russian part of the Curonian Spit. This numbering was used for logistic purposes during the organisation of monitoring activity.

The monitoring network of the State Organization of the Kaliningrad oblast ``Baltberegozaschita'' (BBZ), the local coastal protection authority, was used for more detailed grounding of the found geosynthetic debris. This monitoring network includes reference points with the step of 500 m and covers the whole coastline within the Kaliningrad Oblast. The reference points started at the Polish-Russian border on the Vistula Spit (the point No1 is 500 m north from the Polish-Russian border) and ended at the Lithuanian-Russian border on the Curonian Spit (the point No 289 is just before the Lithuanian-Russian border). All geosynthetic remnants found during the 500 m subsegment were referred to this subsegment (to avoid unnecessary detailing). The subsegments were numbered by the last monitoring reference point of the BBZ monitoring network included in this subsegment.

A preliminary survey [1] showed that fragments of geosynthetic materials are unevenly distributed on the beach. The use of an area-selective technique, such as for anthropogenic debris [2] and microplastics [3], [4], is not resultative in such a case.

**Fig. 2:**
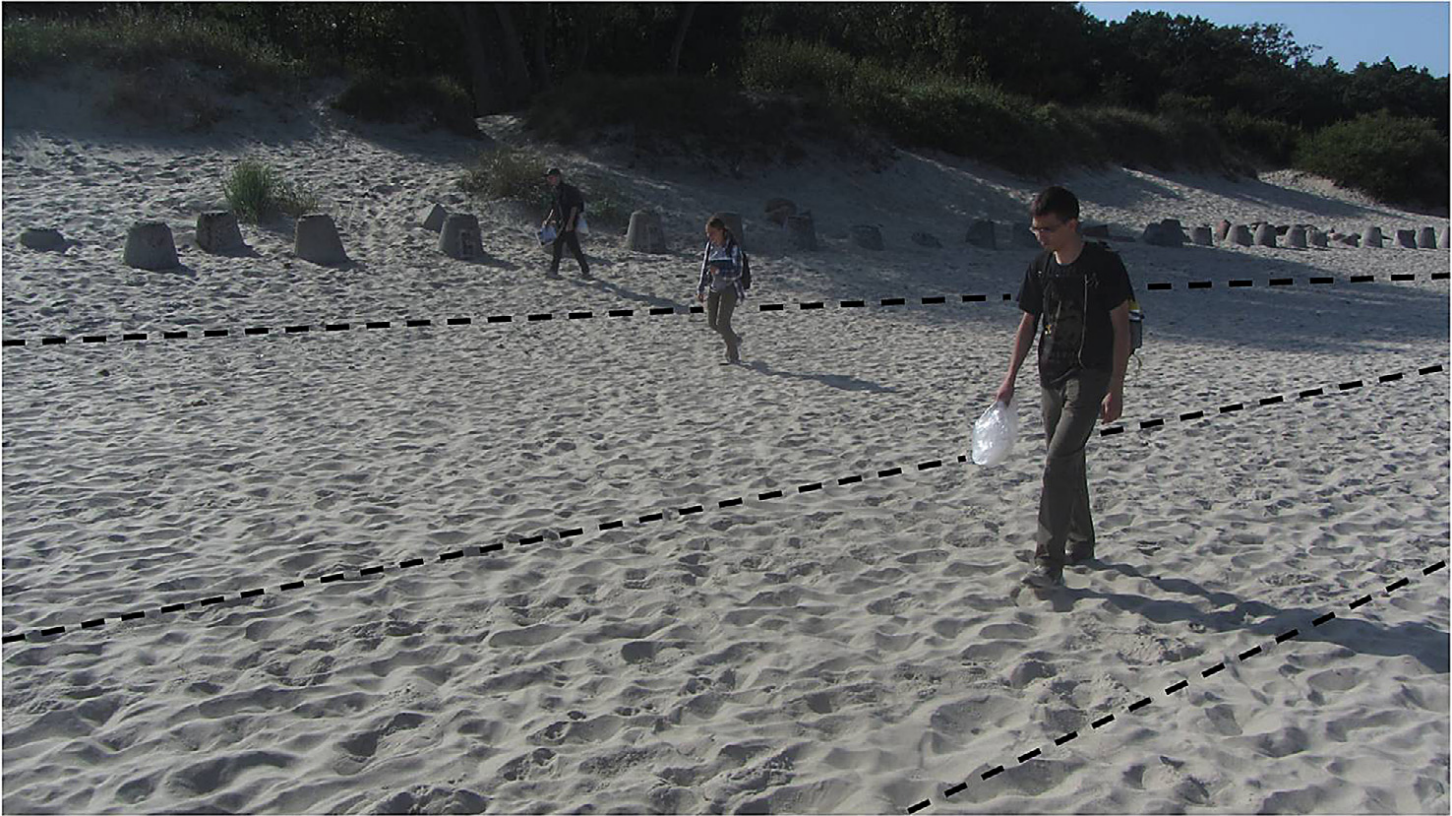
The group of observers on the beach.

The technique of continuous visual scanning [1] has been applied to find the fragments of geosynthetic materials not smaller than meso-forms t(approximately 1 cm in scale). This technique assumes a continuous passage along the entire coastline, covering the entire width of the beach from the edge to the foredune (or cliff), in a group of several observers. The average width of the beaches of the Kaliningrad Oblast is 30 m (up to 190 m in extreme), and the group of observers usually included three people (Fig. 2). The beach (from the coastline to the foredune or cliff) was divided into three control zones; each member of the group controlled the strip of `his'' zone to capture the edge of the neighbouring zone - for a complete scan of the entire beach.

The monitoring has been carried out for three years. In 2018, 29 monitoring visits were carried out in the period from June to November. In 2019, the scope of work amounted to 18 monitoring visits from March to December. The field campaign in 2020 included 13 monitoring visits.

When registering each detected geosynthetic sample, the following parameters were recorded: the type of geosynthetic sample, geometrical dimensions (length and area), number of the subsection where this sample was found, position on the beach (in % of the distance from the waterline, 100% is at the beach back). Next, photographs were taken, and this sample was collected for further laboratory analysis.

The proposed monitoring design helps to assess the beach contamination by geosynthetic debris only superficially. It is impossible to notice all the geosynthetic fragments during a visual inspection of the beach because the fragments:

‐ can be covered with sand or hidden in a heap of pebbles/boulders/algae;
‐ can be smeared or covered with algae or dirt;
‐ do not belong to the types of geosynthetic materials known in advance in the area;
‐ can be severely degraded (destroyed) up to the impossibility of identification;
‐ can be in an inaccessible place (underwater).

Finally, the inattention or fatigue of observers cannot be disregarded.

Various household waste, of which there is a large amount on the beaches, was not considered in this work and was not taken into account. Also, the counts did not take into account tens and hundreds of threads from big bags, which were unevenly distributed along the line of the current splash.

It should be noted that the work was not carried out immediately after the storms passed, and, accordingly, some of the fragments were probably already buried under a layer of sand. Sometimes, the parts of the big bags were partially or almost completely buried in the thickness of the beach, and it did not allow them to be removed from the natural environment and accurately record their sizes.

## Ethics Statement

It is not relevant to this study.

## CRediT author statement

**Boris Chubarenko:** Conceptualisation, Monitoring design development, Methodology development, Organisation of the fieldwork, Reviewing and editing of dataset presentation. Writing – Final version; **Alexander Kileso:** Monitoring design development, organisation of the fieldwork, Field data curation, Preparation of dataset presentation, Visualisation, Statistical assessment, Writing – original draft preparation; **Elena Esiukova:** Methodology development, Field data curation, Development of classification of geosynthetic fragments, Photographing; **Vasiliy Pinchuk:** Field data curation, supervising of the group of observers during the fieldwork; **Franz-Georg Simon:** Reviewing and editing.

## Declaration of Competing Interest

The preparation of the secondary data was organised within the theme 0128-2021-0012 of the State Assignment of the Shirshov Institute of Oceanology of the Russian Academy of Sciences.

The authors declare that they have no known competing financial interests or personal relationships which have or could be perceived to have influenced the work reported in this article.

## Appendix Supplementary materials

Image, application 1

Image, application 2

Image, application 3

Image, application 4

Image, application 5

Image, application 6

Image, application 7

Image, application 8
