# Supplementary material for: Dataset on geosynthetic material debris contamination of the South-East Baltic shore
Source: Data Brief. 2022 Jan 1;40:107778. doi: 10.1016/j.dib.2021.107778 (PMC8741436; doi:10.1016/j.dib.2021.107778)
Supplement: Supplementary file 1 [file mmc1.pdf]

1. List of types of geosynthetic material residues

This section contains information about types of geosynthetic material debris found on the shore of the Kaliningrad Region (Russian, South-East Baltic) during field surveys in the 2018 - 2020 ERANET-RUS_Plus joint project EI-GEO, ID 212 (RFBR 18-55-76002 ERA_a, BMBF 01DJ18005).

Geosynthetic materials are made from polypropylene (PP), polyester (PET), polyethylene (PE), high-density polyethylene (HDPE), polyamide (nylon), polyvinyl chloride (PVC), and fibreglass. PP and PET are the most widely used materials.

The most frequent found debris of geosynthetic materials are related to four types: geotextile, degraded gabion coating, geocontainers and geocells (see figures further).

1.1. Samples of the geotextile materials.

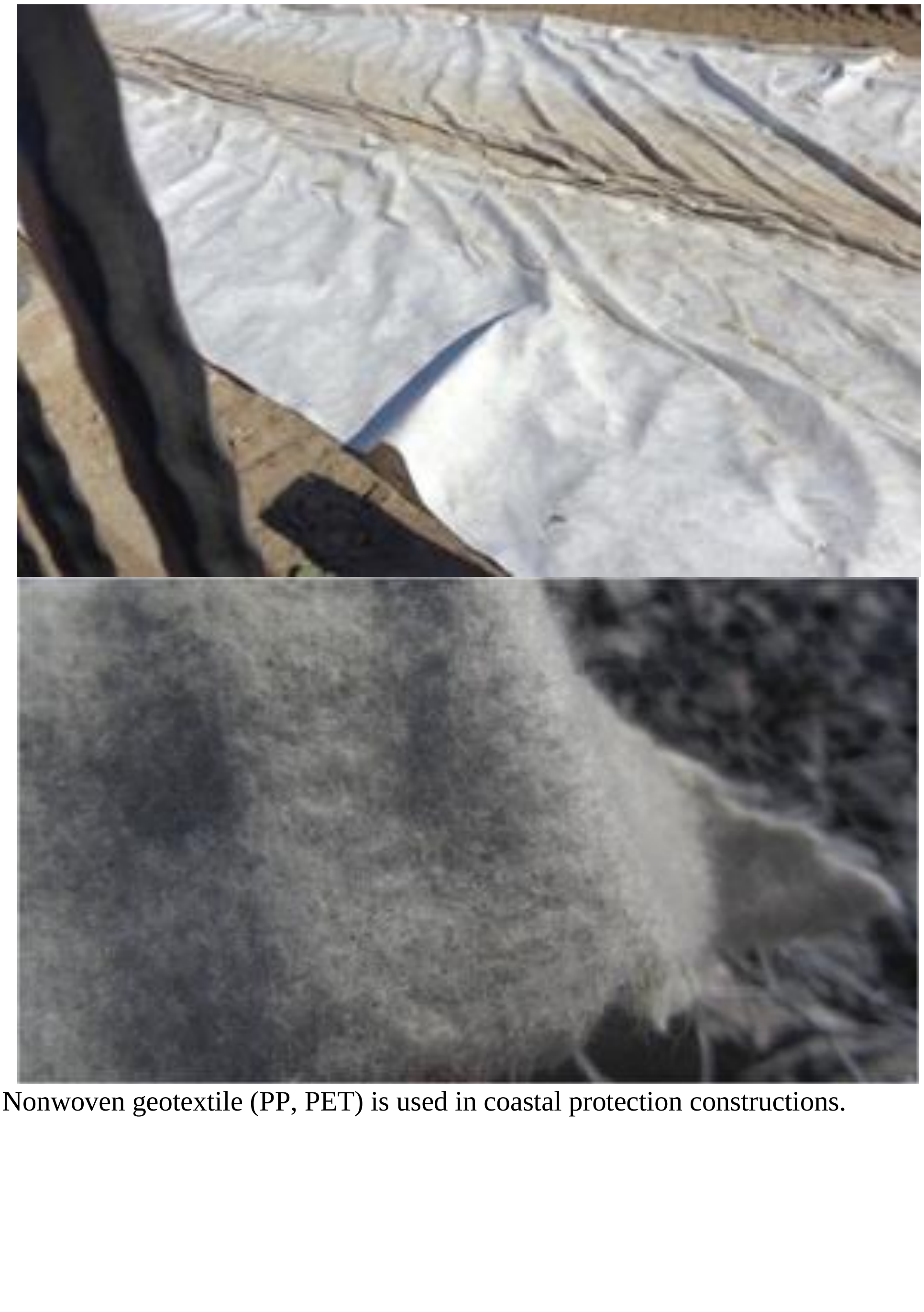

Figure 1.1. Nonwoven geotextile (PP, PET) is used in coastal protection constructions.

.

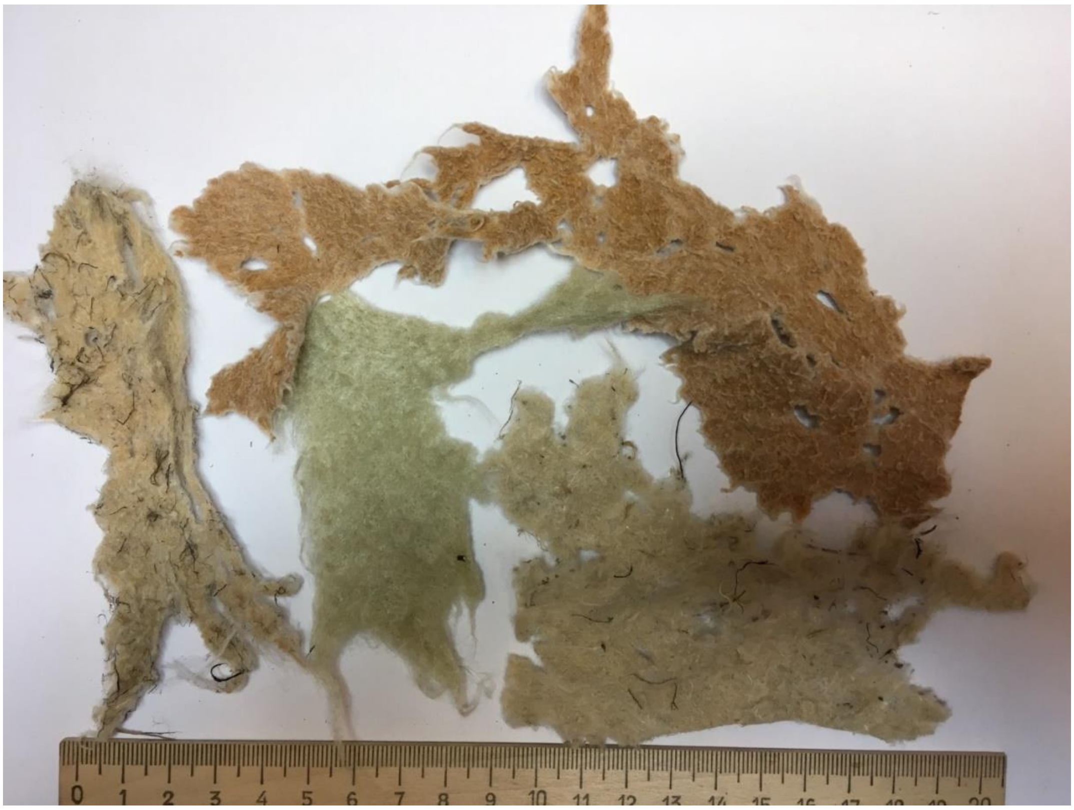

Figure 1.2. Fragments of nonwoven geotextile (PP, PET). All pieces are not fresh; they were a long time in natural conditions.

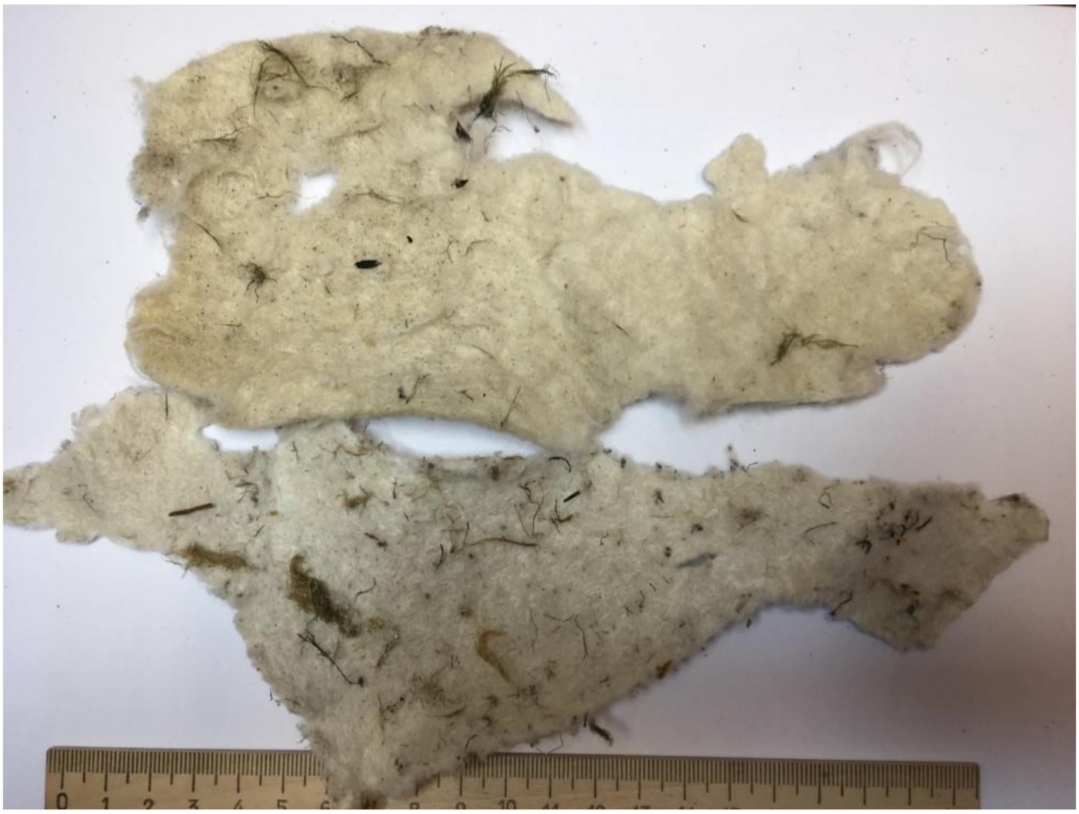

Figure 1.3. Fragments of white nonwoven geotextile (PP, PET). Pieces are not fresh; they were a long time in natural conditions.

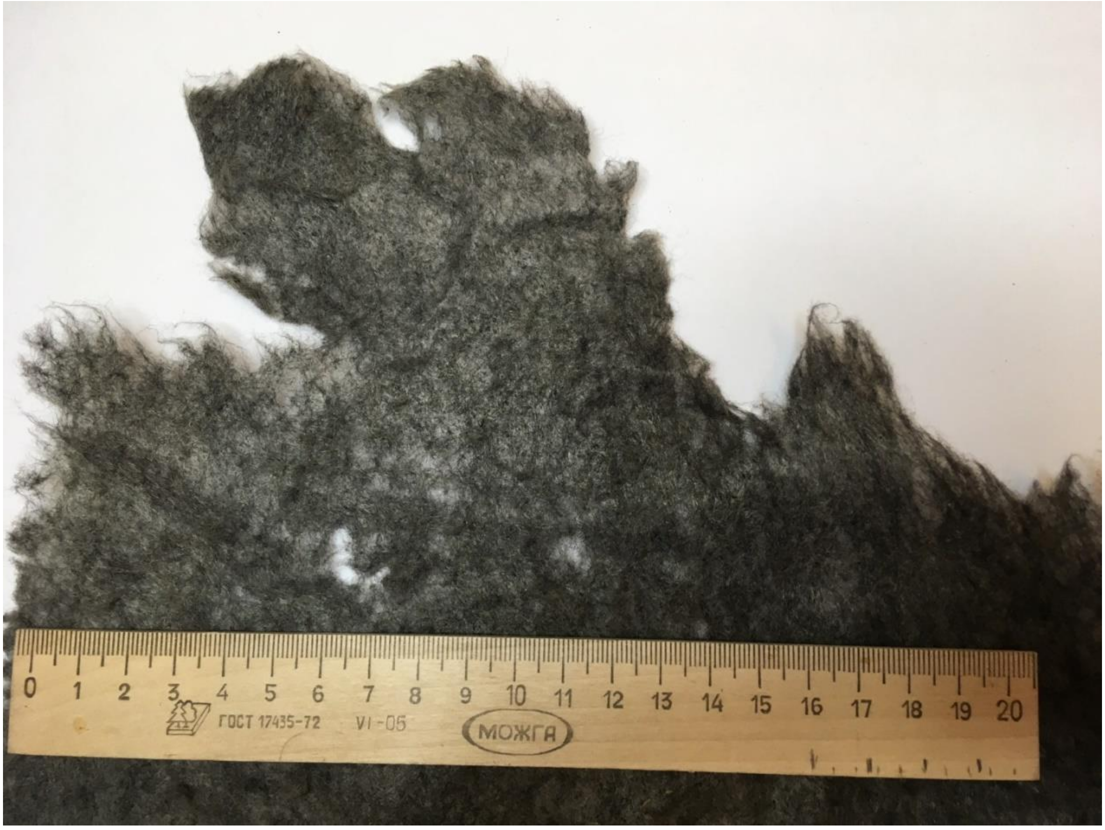

Figure 1.4. The Fragment of black nonwoven geotextile (PP, PET)). Pieces are not fresh; they were a long time in natural conditions.

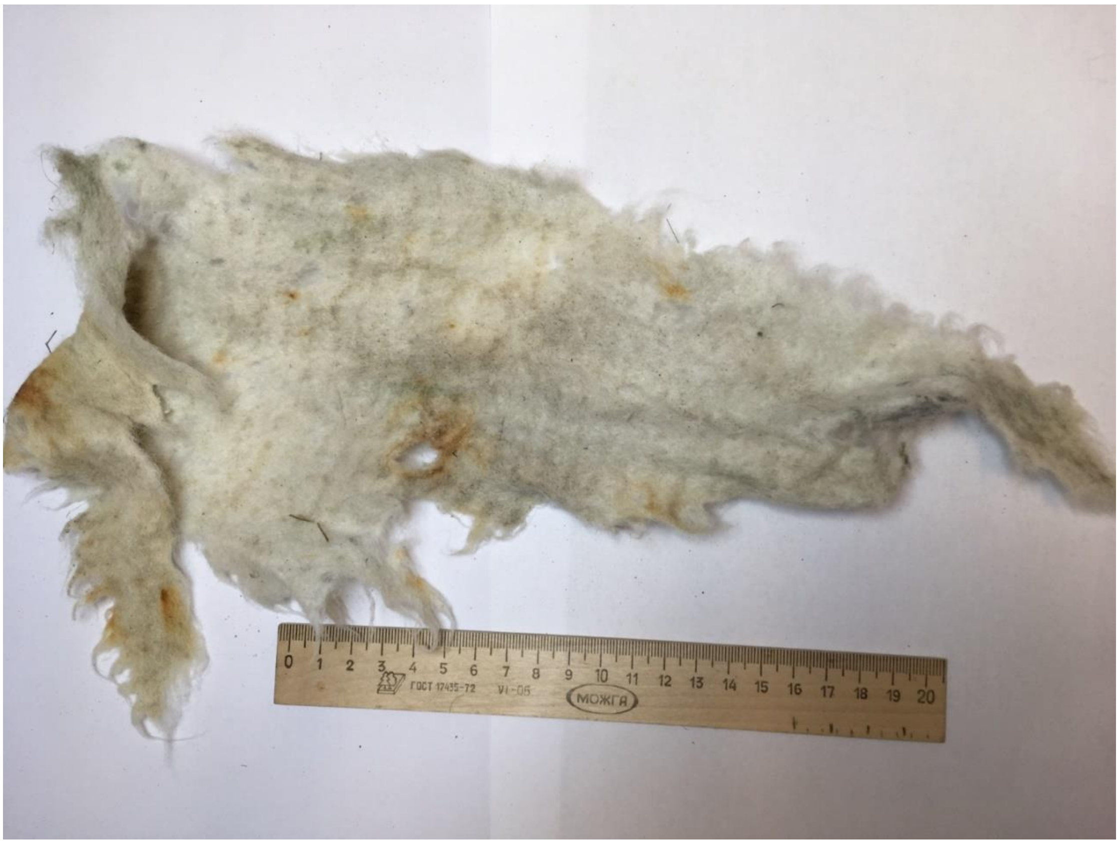

Figure 1.5. The Fragment of white nonwoven geotextile (PP, PET) with reinforcing stitching. The pieces is not fresh; it was a long time in nature.

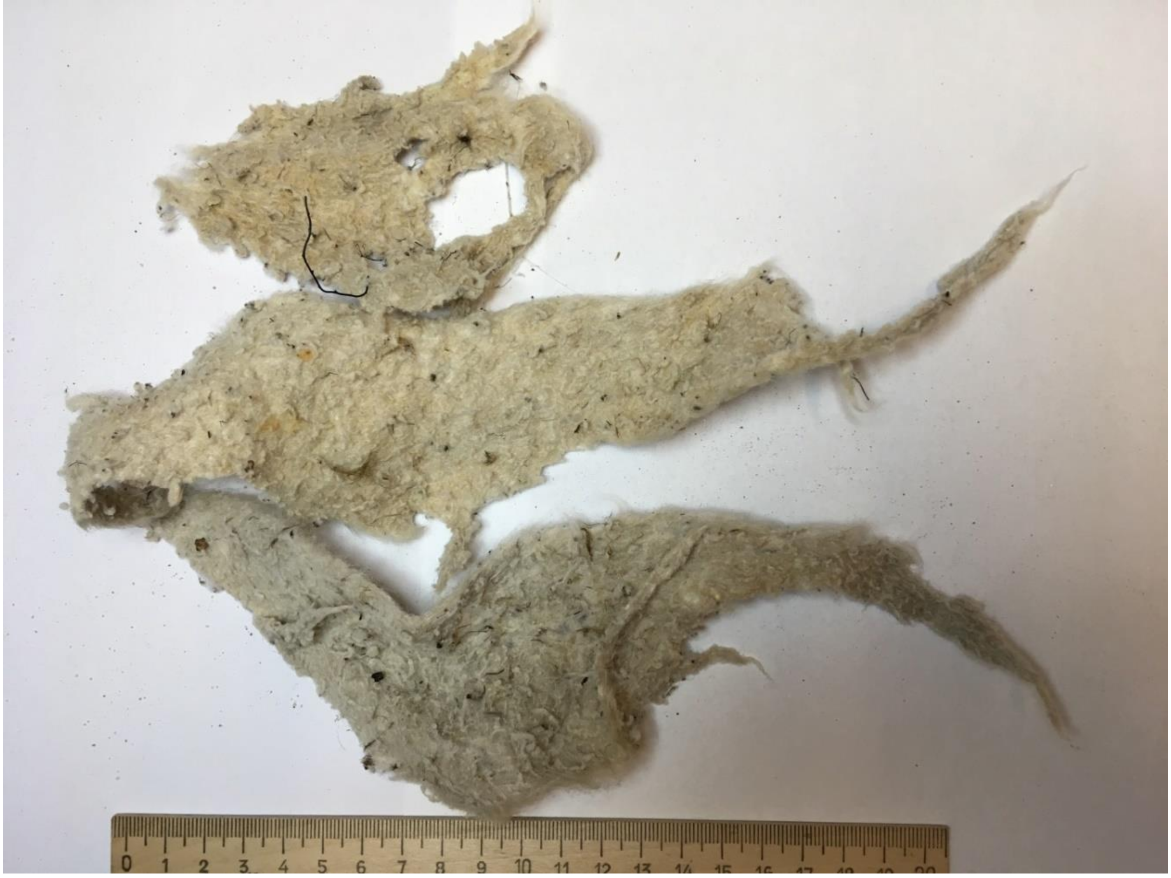

Figure 1.6. The Fragment of white nonwoven geotextile (PP, PET)). Pieces are not fresh; they were a long time in natural conditions.

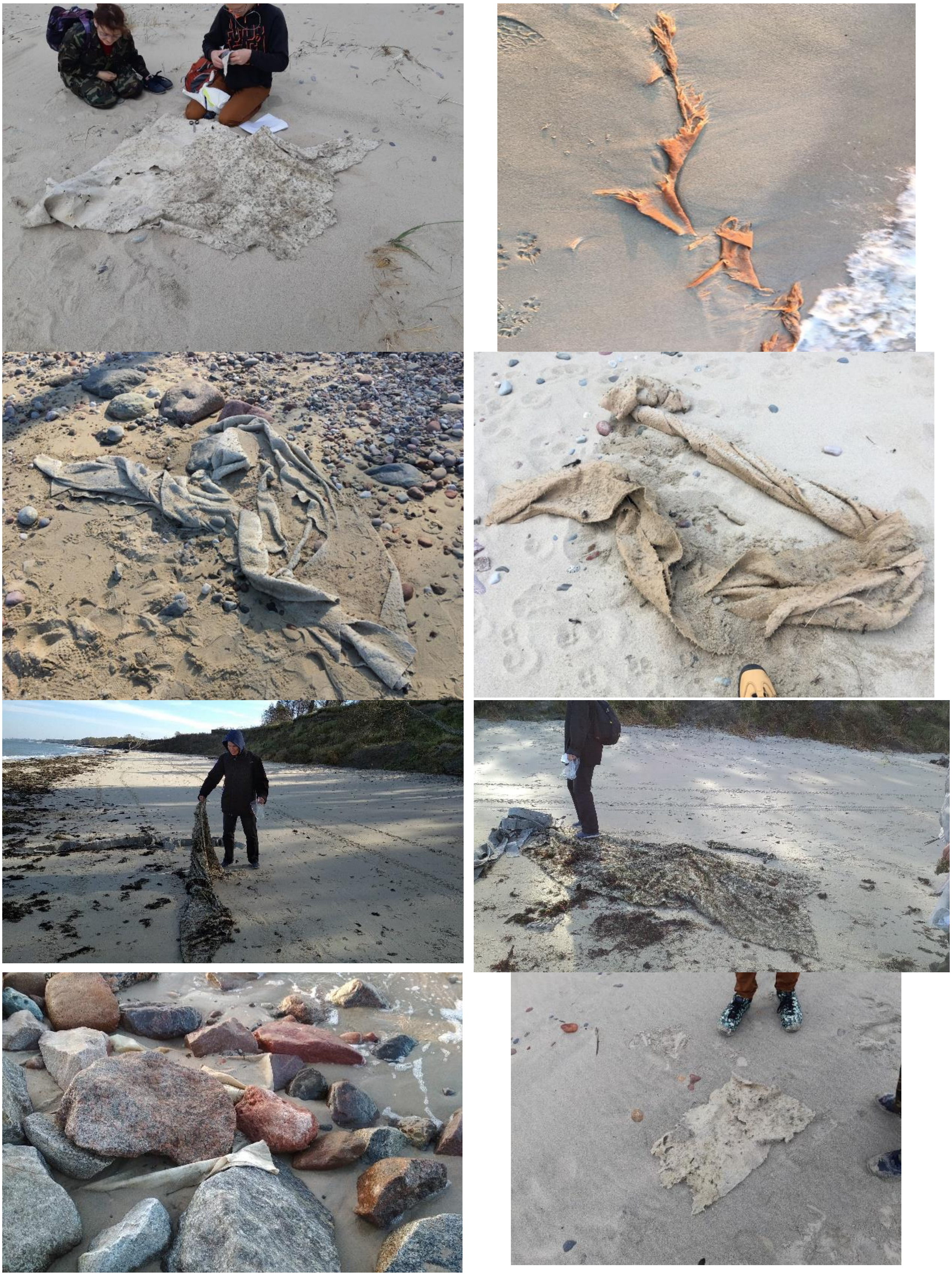

Figure 1.7. Examples of the geotextile debris on the beaches of the Kaliningrad Oblast, Russia.

1.2. Samples of the degraded gabion coating

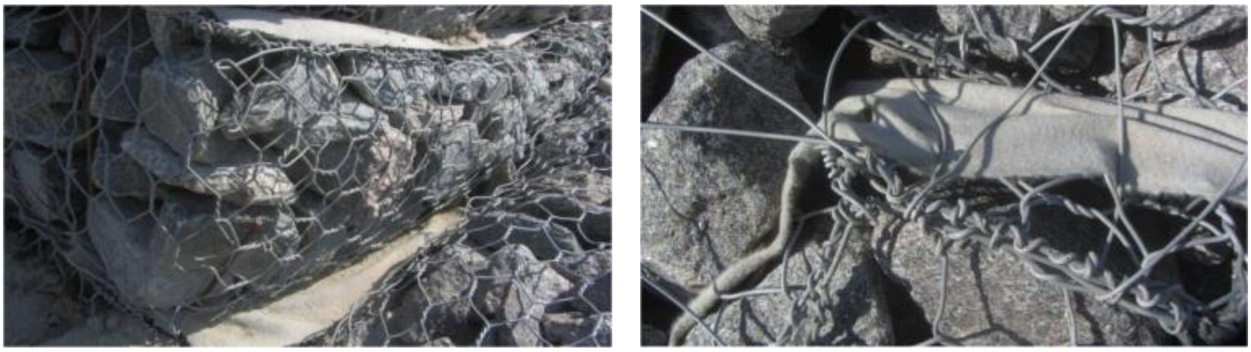

Figure 1.8. Gabion wire braid.

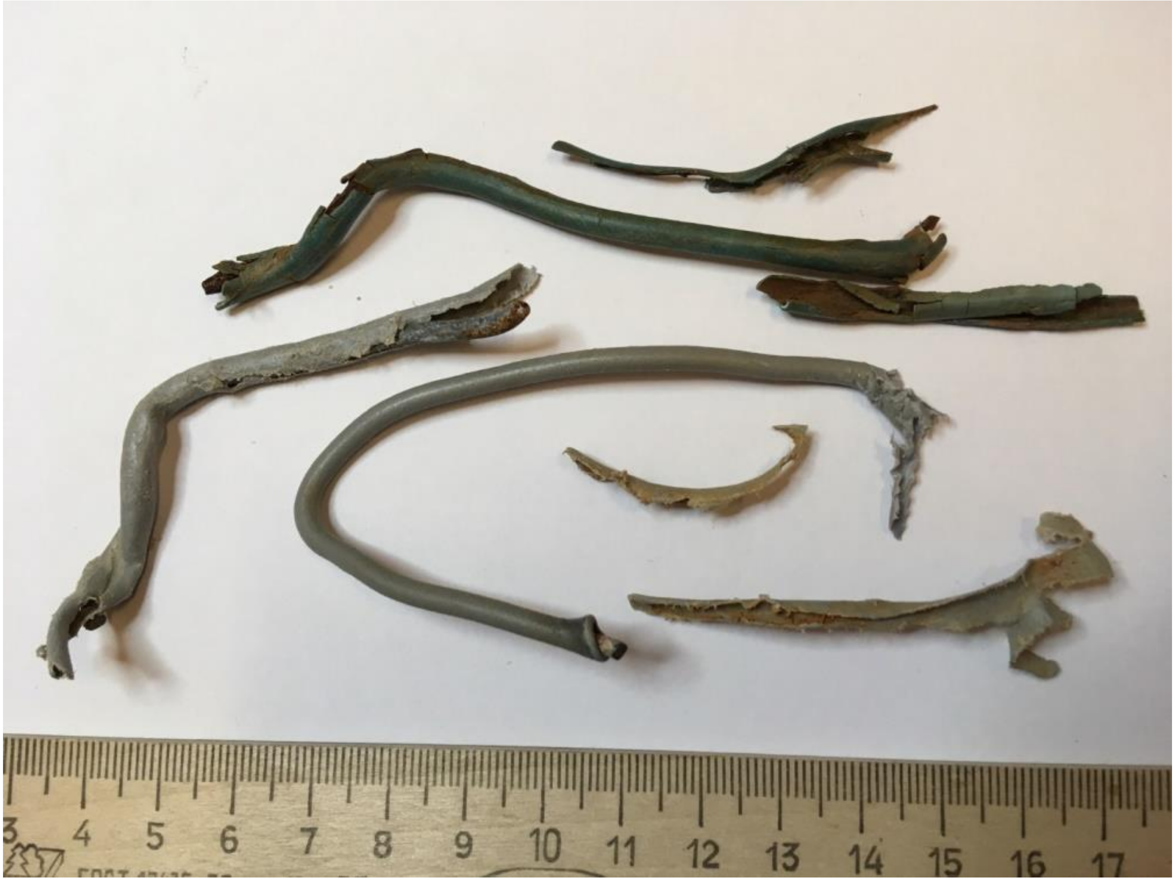

Figure 1.9. Usually, only fragments of plastic coating for gabion wire are present on the beach, but sometimes the pieces contain the wire (two pieces in the figure).

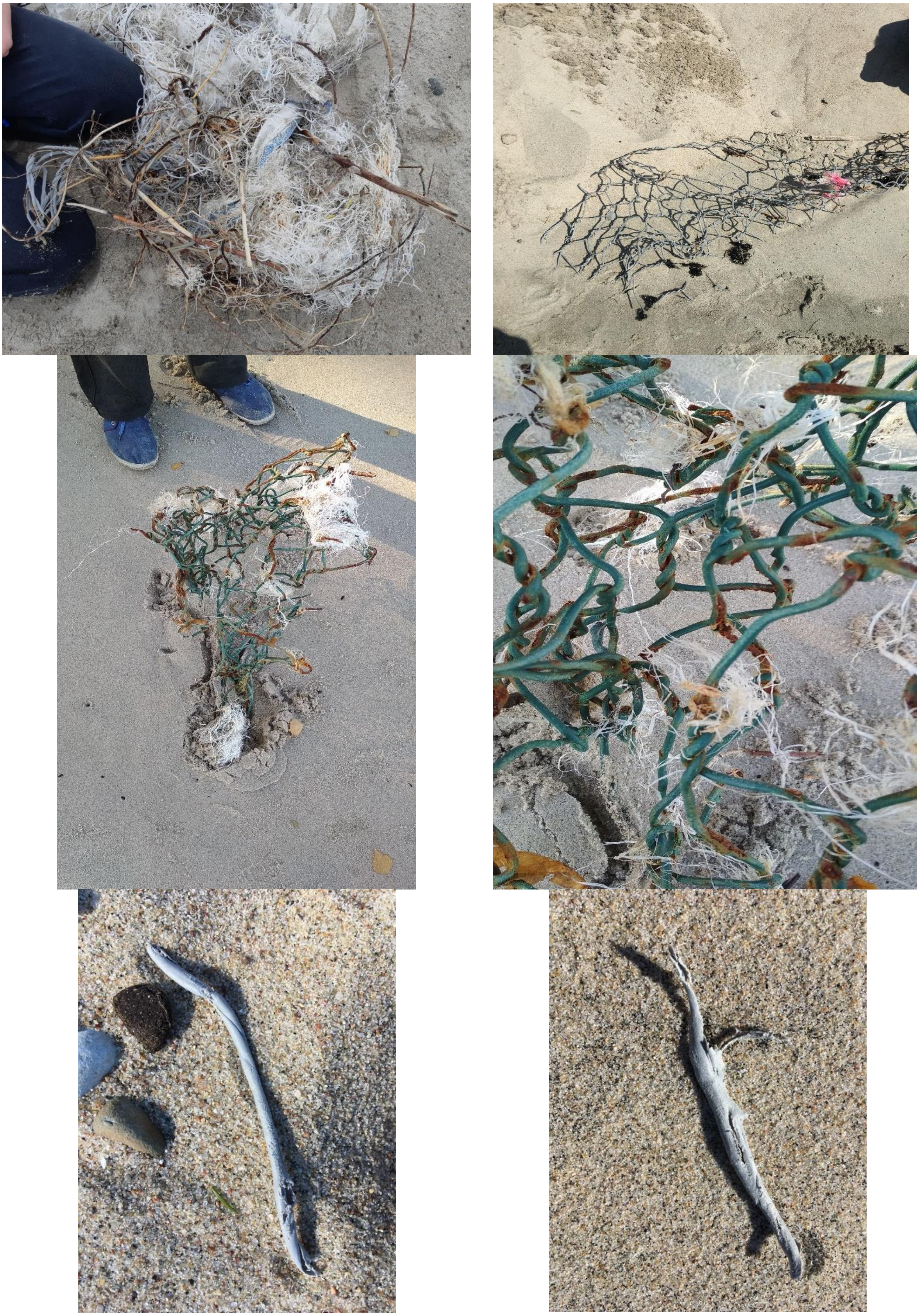

Figure 1.10. Examples of the gabion fragments on the beaches of the Kaliningrad Oblast, Russia.

1.3. Samples of the debris of the geocontainers

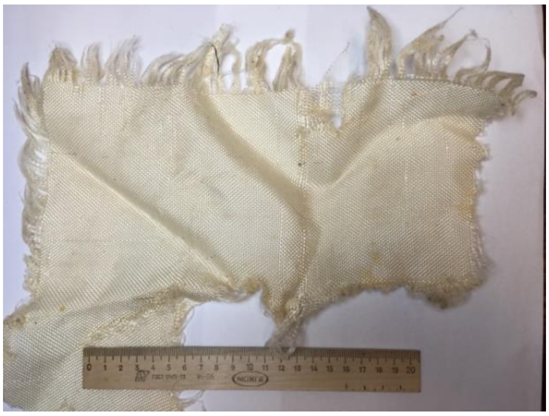

(a)

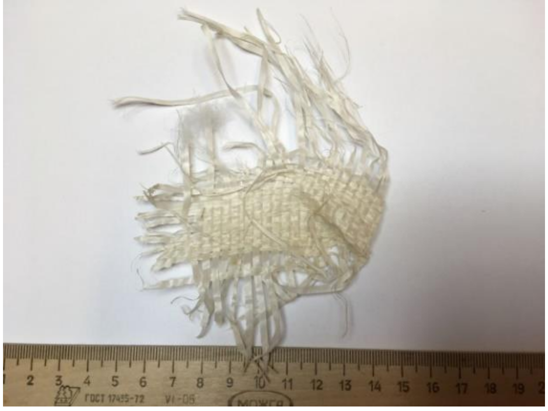

(b)

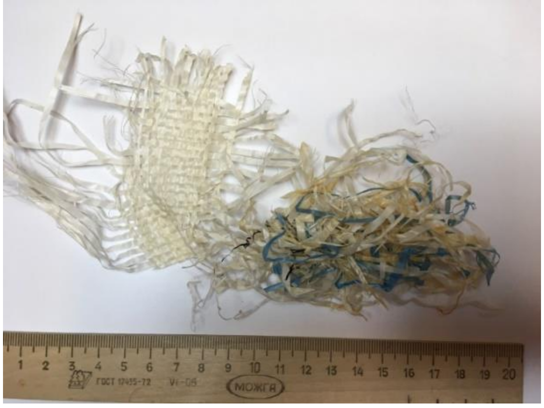

(c)

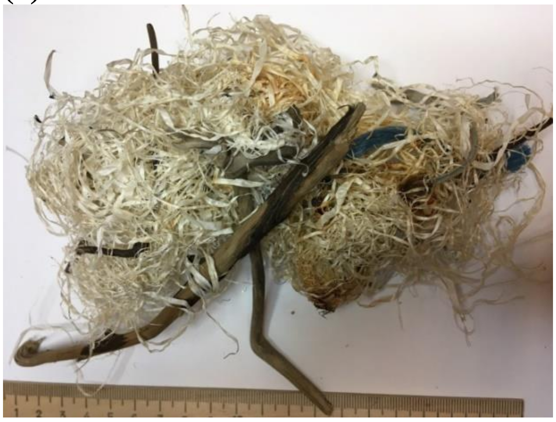

(d)

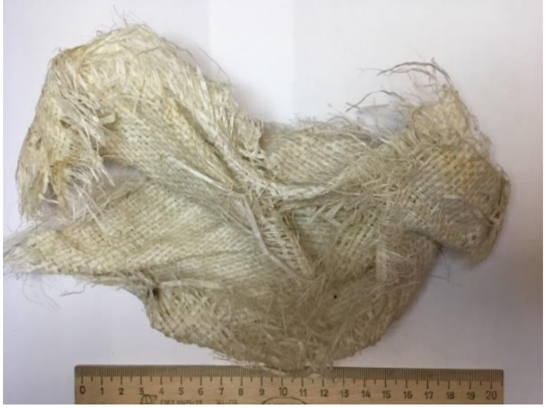

(e)

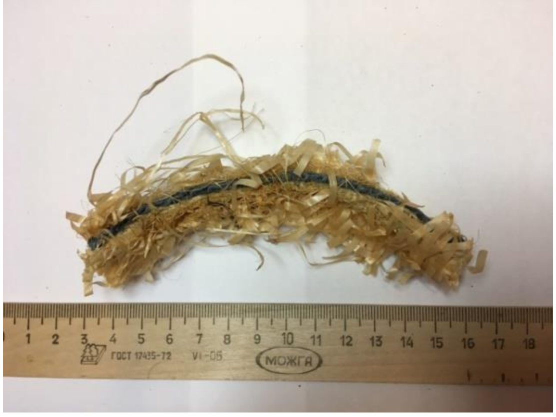

(f)

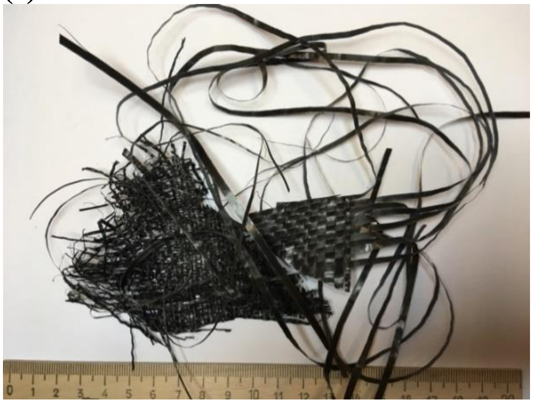

(g)

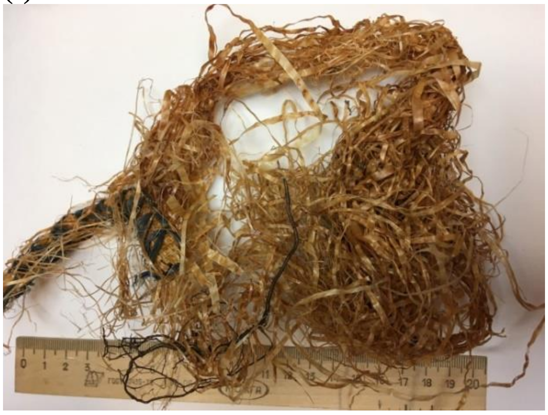

(h)

Figure 1.11. Fragments of woven material.

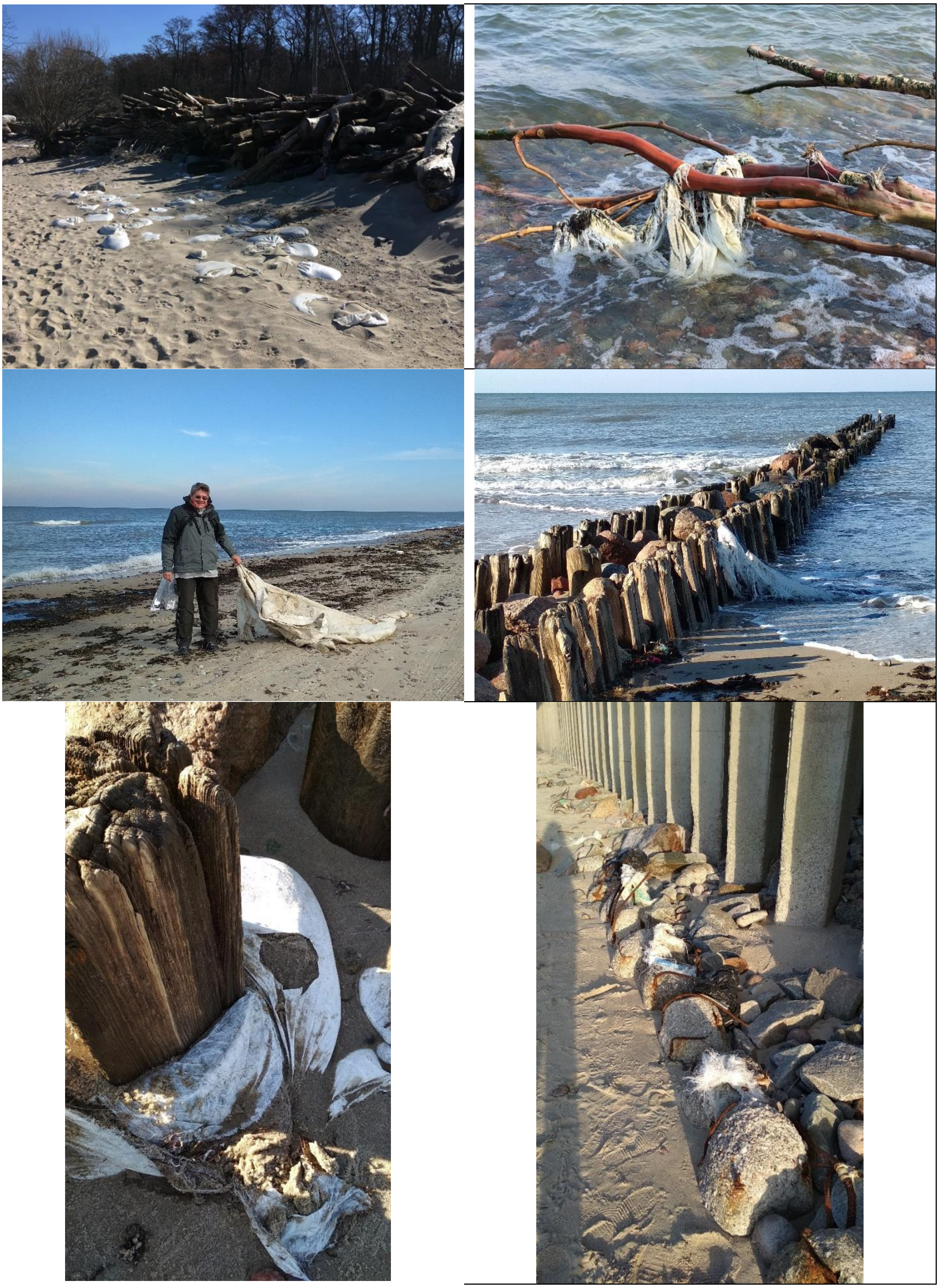

Figure 1.12. Examples of the geocontainer fragments on the beaches of the Kaliningrad Oblast, Russia.

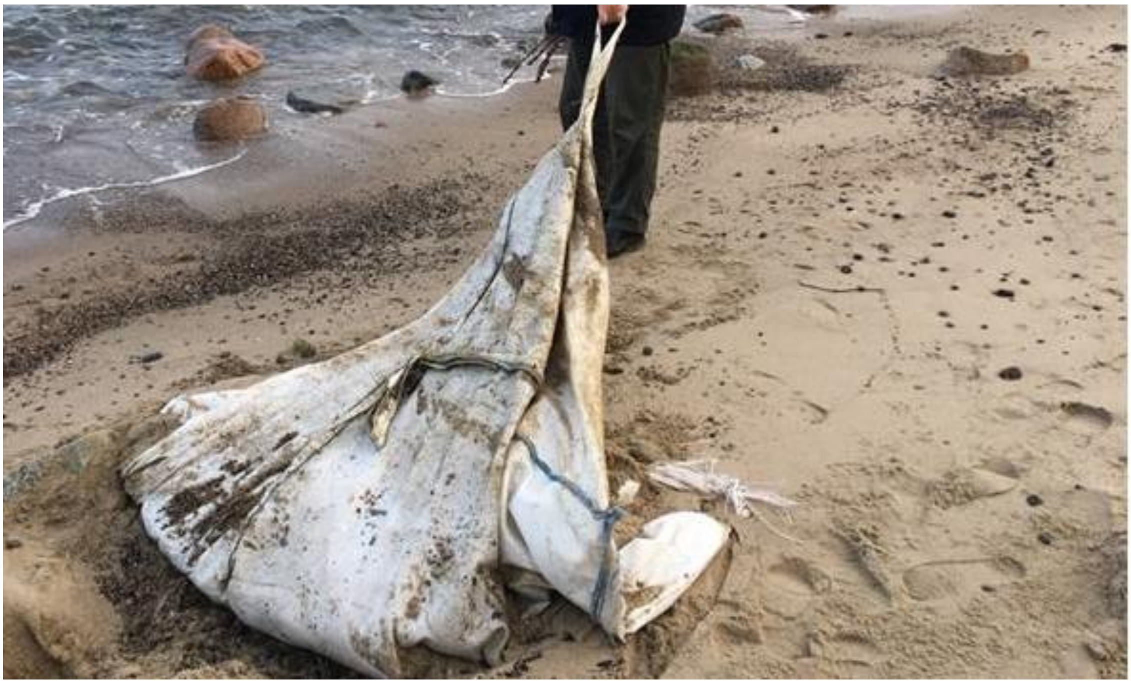
Figure 1.13.Used geocontainer (a woven bag, HDPE, PP).

1.4. Samples of the debris of the geocells.

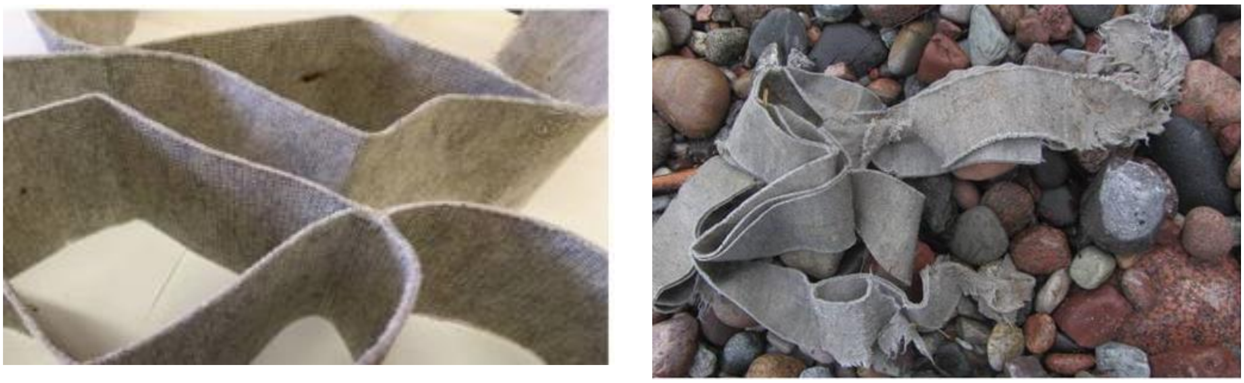

Figure 1.14. Geocells are made from PP, HDPE or PE fibres.

(a) 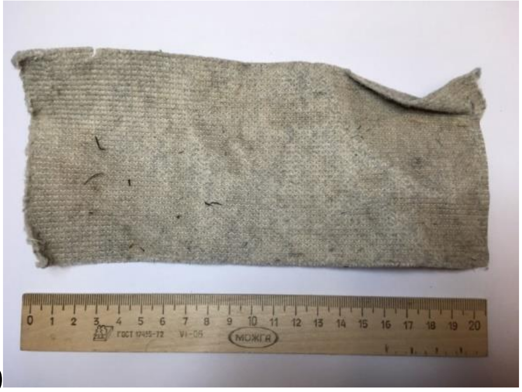

(b) 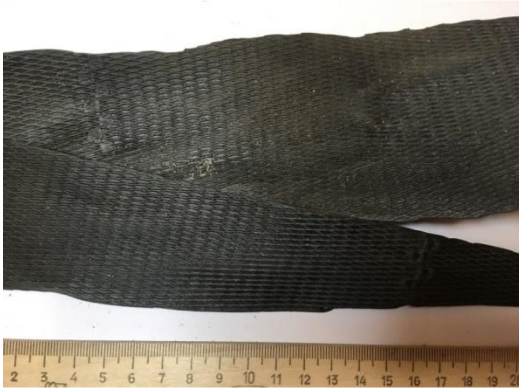

(c) 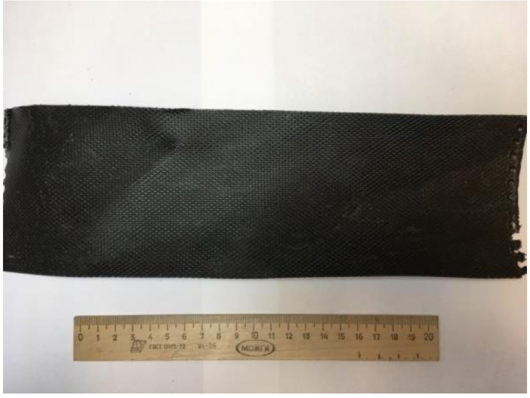

Figure 1.15. Fragments of the tape from which geocells are produced.

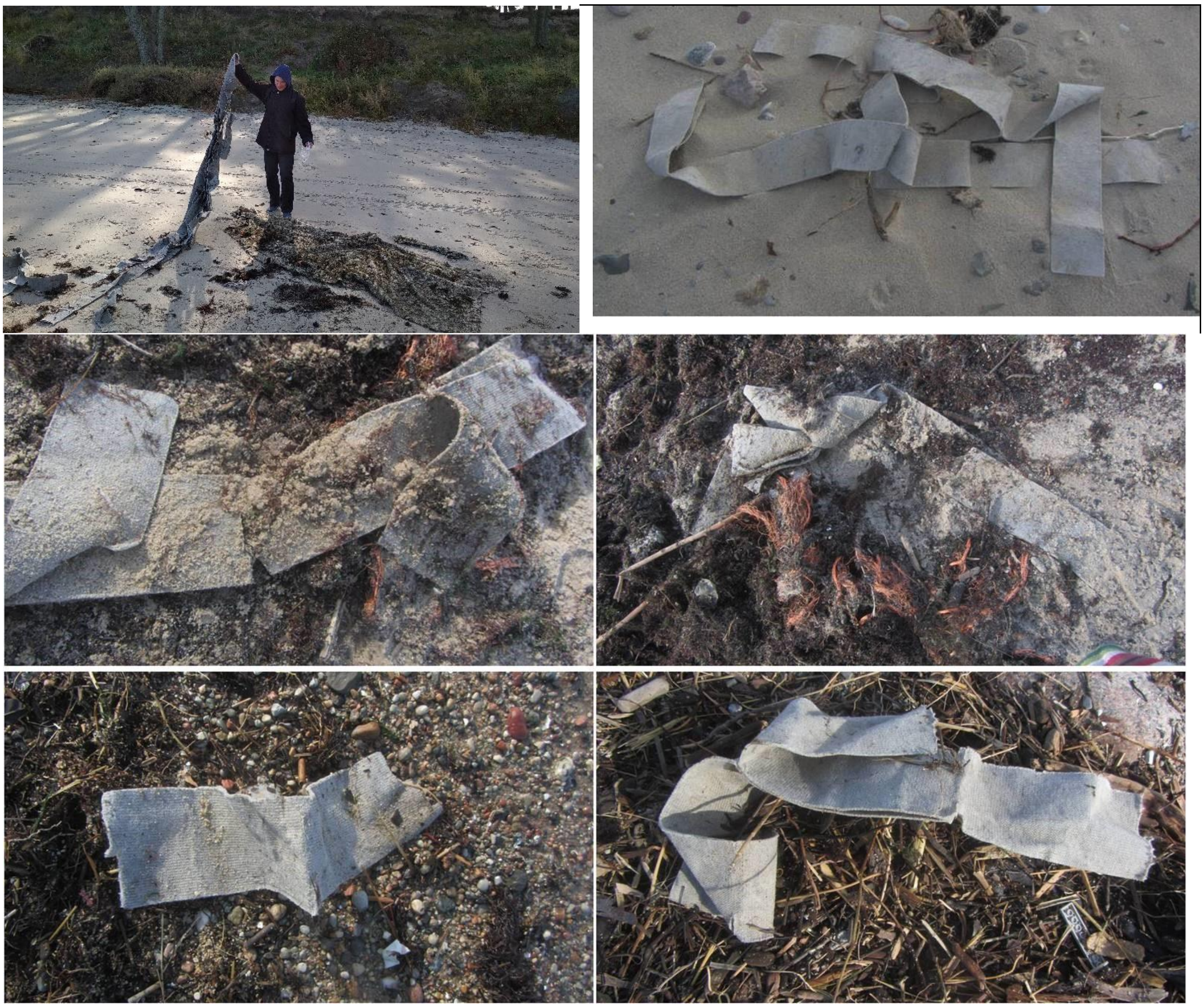

Figure 1.16. Examples of the geocell fragments on the beaches of the Kaliningrad Oblast, Russia.

1.5. Samples of the debris of the geomats.

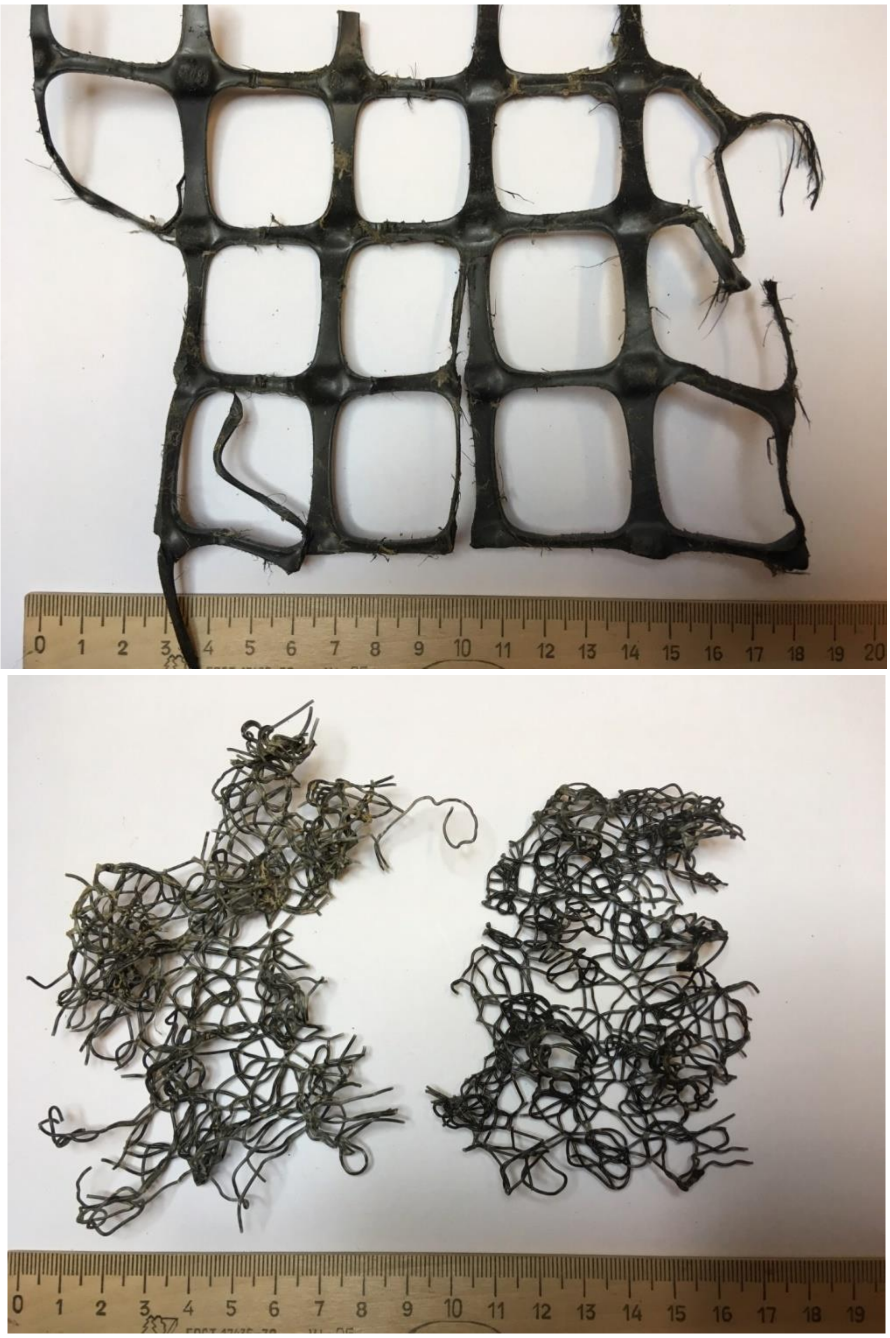

(a)

(b)

Figure 1.17. Fragments of the geomat.
