# Supplementary material for: Dataset on geosynthetic material debris contamination of the South-East Baltic shore
Source: Data Brief. 2022 Jan 1;40:107778. doi: 10.1016/j.dib.2021.107778 (PMC8741436; doi:10.1016/j.dib.2021.107778)
Supplement: Supplementary file 6 [file mmc6.pdf]

3. Statistics for geosynthetic debris found on the shore of Kaliningrad Oblast (2018-2020)

This section contains information about statistics on sample size (geometrical dimensions: length and area) for different types of geosynthetic material debris found on the shore of the Kaliningrad Oblast (Russian, South-East Baltic) during field surveys in the 2018 - 2020 ERANET-RUS_Plus joint project EI-GEO, ID 212 (RFBR 18-55-76002 ERA_a, BMBF 01DJ18005).

The statistics on sample size are presented in the form of a box-and-whisker diagram for debris of geotextile (Fig.3.1), geocontainer (Fig.3.2) and gabion plastic coating (Fig.3.3) for each monitoring year. For geotextile and geocontainer, variations of a sample area (cm^2) are presented (Figs 3.1 and 3.2), while for gabions, the variations of a sample length (cm) are presented (Fig. 3.3).

The diagrams were not prepared for other types of geosynthetic materials (geocells, geomats) due to the small number of collected samples. The samples usually have such a complicated geometry. The dimensions of the samples were estimated and rounded.

On each box-and-whisker diagram, the label inside the box indicates the value of the median sample size. Upper and lower whiskers correspond to the maximum and minimum values. The upper whiskers are also labelled.

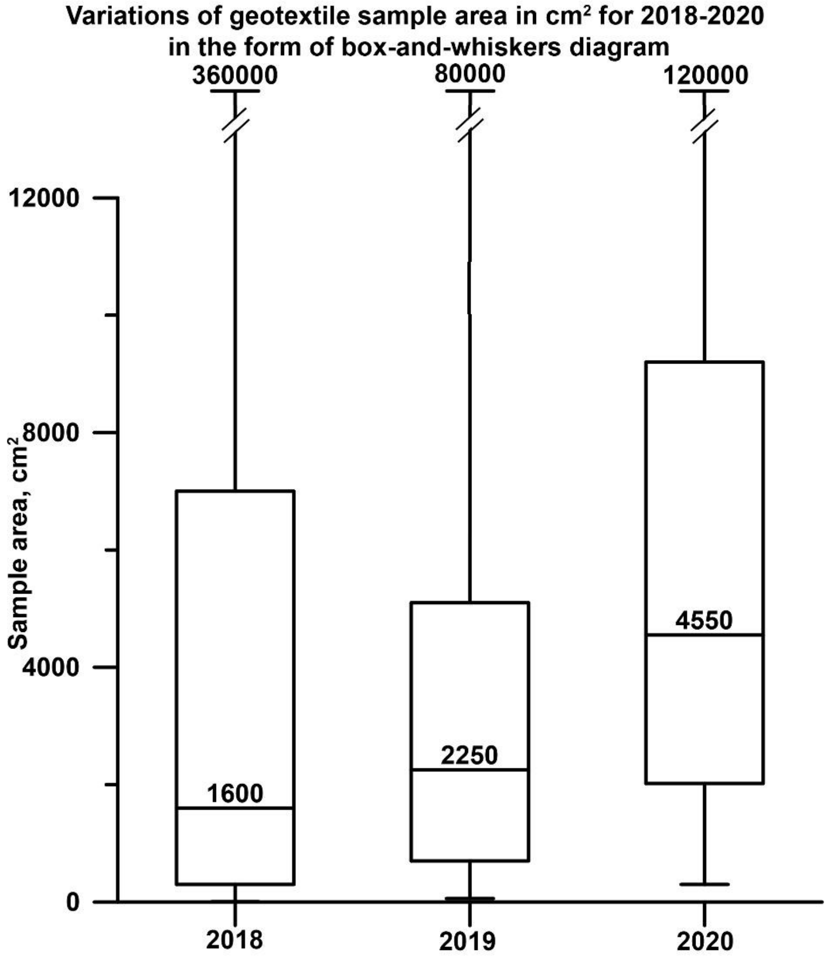

Figure 3.1. Variation of geotextile sample area in cm^2 for 2018-2020 in the form of a box-and-whiskers diagram. The label inside the box is the median value, the label above the upper whisker is the maximum value.

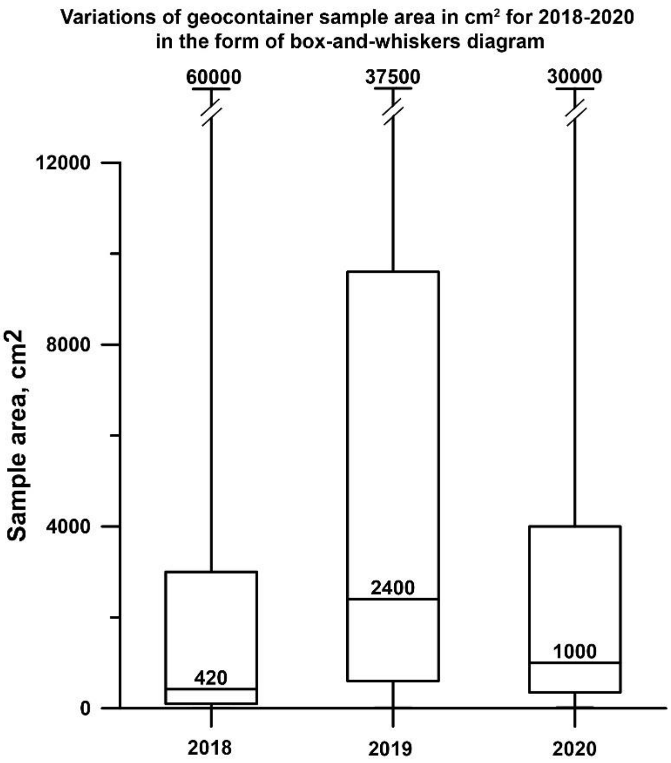

Figure 3.2. Variation of geocontainer sample area in cm^2 for 2018-2020 in the form of a box-and-whiskers diagram. The label inside the box is the median value, the label above the upper whisker is the maximum value.

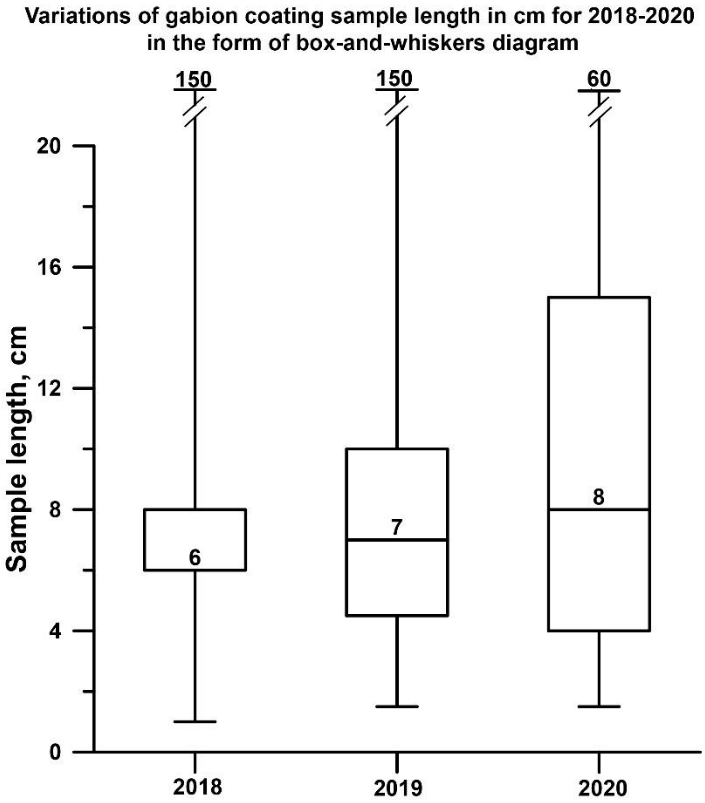

Figure 3.3. Variation of gabion plastic coating sample length in cm for 2018-2020 in the form of a box-and-whiskers diagram. The label inside the box is the median value, the label above the upper whisker is the maximum value.
