# Supplementary material for: Dataset on geosynthetic material debris contamination of the South-East Baltic shore
Source: Data Brief. 2022 Jan 1;40:107778. doi: 10.1016/j.dib.2021.107778 (PMC8741436; doi:10.1016/j.dib.2021.107778)
Supplement: Supplementary file 7 [file mmc7.pdf]

4. Results of the test surveys on the shores of Lithuania and Poland adjacent to Kaliningrad Oblast

This section contains information about types of geosynthetic material debris found on the shore of the Polish and Lithuanian coasts adjacent to Kaliningrad Oblast (South-East Baltic) during field surveys in May-June 2019 within the ERANET-RUS_Plus joint project EI-GEO, ID 212 (RFBR 18-55-76002 ERA_a, BMBF 01DJ18005).

There were twelve test 1-km segments at the Lithuanian part of the shore of the Southeastern Baltic (Fig. 4.1a): six 1-km segments on the Lithuanian part of the Curonian Spit (spit sector) and six segments on the mainland to the north towards the Latvian-Lithuanian border (mainland sector).

Seven test segments of various lengths were defined at the Polish part of the neighbouring shore - five segments on the Polish part of the Vistula Spit and two segments between the Vistula River mouth and the core of the Vistula spit (Fig.4.1b).

Information about field surveys is in Table 4.1. Results are in Table 4.2.

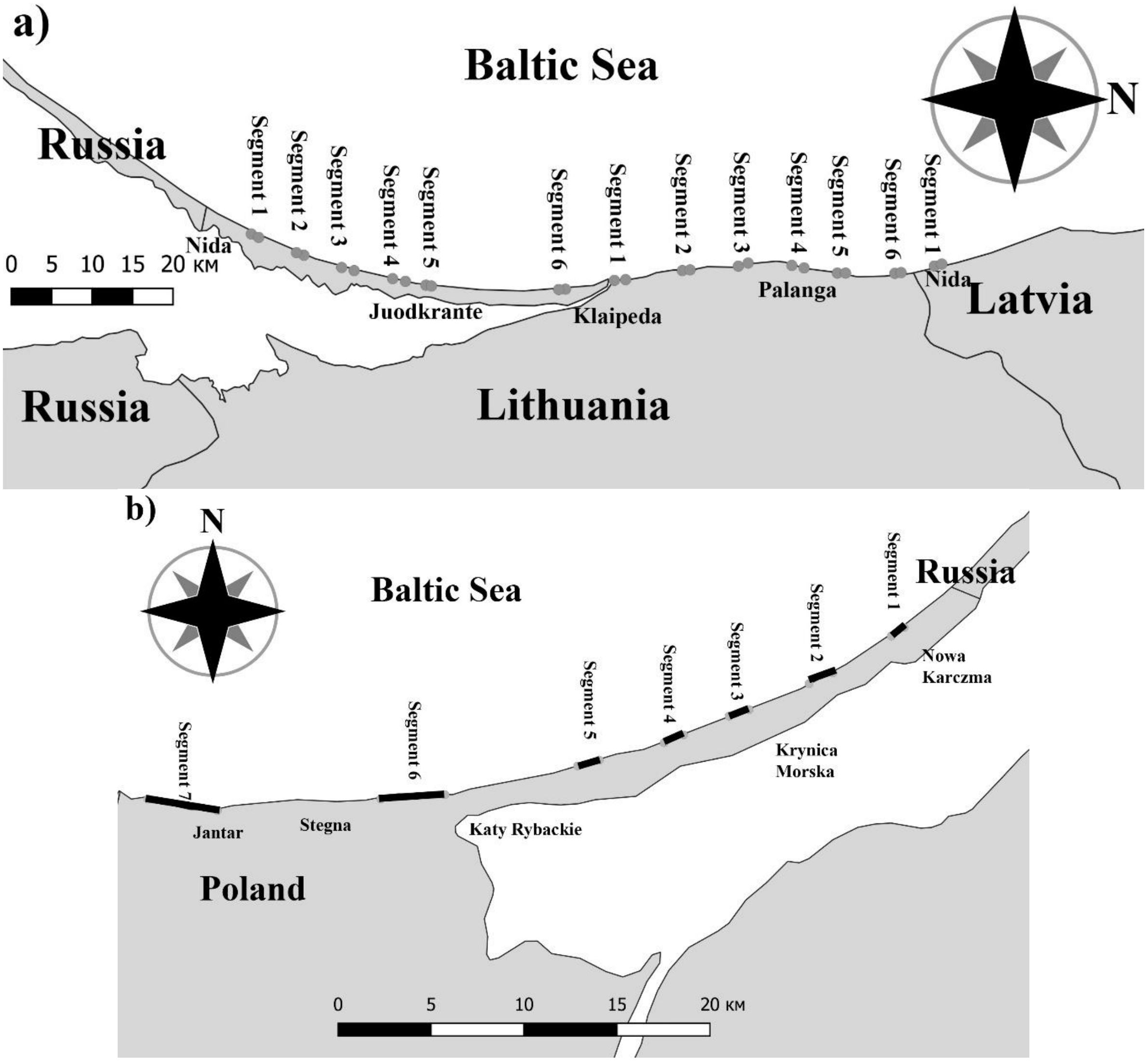

Figure 4.1. Positionsa of the monitoring segments on the Lithuanian (a) and Polish (b) shores.

Table 4.1. Information about test field surveys on the shore of the Lithuanian and Polish coasts adjacent to Kaliningrad Oblast (South-East Baltic) in May-June 2019 within the ERANET-RUS_Plus joint project EI-GEO, ID 212 (RFBR 18-55-76002 ERA_a, BMBF 01DJ18005).

	Segment number	Coordinates South [Lon_Lat_degree]	Coordinates North [Lon_Lat_degree]	Length [km]	Time survey [month, year]
Lithuania (Guronian Spit)	1	20.99445 55.32765	21.00111 55.33583	1	May 2019
	2	21.03583 55.38611	21.03083 55.37778	1	May 2019
	3	21.05972 55.42806	21.06583 55.44194	1.6	May 2019
	4	21.08167 55.48472	21.08694 55.49889	1.6	May 2019
	5	21.09444 55.52167	21.09611 55.52778	0.8	May 2019
	6	21.10278 55.67667	21.10333 55.66917	1	May 2019
Lithuania (mainland)	1	21.08555 55.73111	21.08416 55.74361	1.5	May 2019
	2	21.06638 55.80666	21.06500 55.81527	1	May 2019
	3	21.05777 55.86888	21.05166 55.87972	1.5	May 2019
	4	21.05638 55.92833	21.06138 55.94194	1.6	May 2019
	5	21.07166 55.97888	21.07166 55.98805	1	May 2019
	6	21.07222 56.04277	21.07083 56.04972	0.9	May 2019
Latvia	1	21.05777 56.08666	21.05388 56.09500	1	May 2019
Poland	1	54.43944 19.60166	54.43500 19.59055	1	June 2019
	2	54.41638 19.54222	54.41055 19.52444	1.5	June 2019
	3	54.39555 19.47416	54.39555 19.47416	1	June 2019
	4	54.38222 19.42222	54.37972 19.40750	1.1	June 2019
	5	54.37972 19.34666	54.36694 19.33333	1.5	June 2019
	6	54.34666 18.97694	54.34388 19.03472	3.5	June 2019
	7	54.35000 19.16972	54.35305 19.22277	3.8	June 2019

Table 4.2. Number of geosynthetic material debris which was found on Lithuanian and Polish coasts adjacent to Kaliningrad Oblast (South-East Baltic) during field surveys in May-June 2019

	Segment number	Geotextile [numbers]	Gabion coating [numbers]	Geocontainer [numbers]	Geocell [numbers]	Geomat [numbers]
Lithuania (Guronian Spit)	1	0	1	3	0	0
	2	1	1	1	0	0
	3	1	2	15	0	0
	4	0	4	8	0	1
	5	0	1	0	0	0
	6	0	0	3	0	0
Lithuania (mainland)	1	0	2	1	0	0
	2	0	0	2	0	0
	3	0	1	2	0	0
	4	0	2	0	0	0
	5	0	0	0	0	0
	6	0	0	0	0	0
Latvia	1	0	0	0	0	0
Poland	1	0	0	0	0	0
	2	0	0	0	0	0
	3	0	0	0	0	0
	4	0	0	0	0	0
	5	0	0	0	0	0
	6	0	4	0	0	0
	7	0	28	0	0	0
