# Supplementary material for: Dataset on geosynthetic material debris contamination of the South-East Baltic shore
Source: Data Brief. 2022 Jan 1;40:107778. doi: 10.1016/j.dib.2021.107778 (PMC8741436; doi:10.1016/j.dib.2021.107778)
Supplement: Supplementary file 8 [file mmc8.pdf]

Debris of geosynthetic materials on the shore of the South-Eastern Baltic (Kaliningrad Oblast, the Russian Federation)

E. E. Esiukova[1], B. V. Chubarenko[1], F.-G. Simon[2]
[1] Shirshov Institute of Oceanology, Russian Academy of Sciences,
36, Nahimovskiy prospekt, Moscow, Russia, 117997
[2] The Federal Institute for Materials Research and Testing, Unter den Eichen 87, 12205 Berlin, Germany
elena_esiukova@mail.ru

Geosynthetics are widely used in hydraulic engineering and within coastal protection constructions at the Baltic Sea shore, such as walls, promenades, and gabions walls. Storms influence leads to deformation of some of the protection structures and cause the release of geotextiles onto the beach. Fragments of geotextile migrate along the shore, experiencing additional degradation and destruction down to macro-, meso-, and micro-particles. During October 2017 - March 2018, the Baltic Sea shore along the Sambia Peninsula (Kaliningrad Oblast of the Russian Federation) was monitored to establish the contamination of sandy beaches by geotextiles that had degraded. Several local sources of pollution of beaches by geosynthetic materials were established.

I. Introduction

According to [1] there are 9 main categories of the geosynthetics: (1) geotextiles, (2) geogrids, (3) geonets, (4) geomembranes, (5) geosynthetic clay liners, (6) geofoam, (7) geocells, (8) drainage / infiltration cells, and (9) geocomposites. Each category is widely used in hydraulic engineering, in coastal protection and coastal engineering design [2]. Geotextiles are any textile materials used in the soil for technical purposes, or "any permeable textile material used for filtration, drainage, separation, reinforcement and stabilization purposes as an integral part of civil engineering structures of earth, rock or other constructional materials" [3, 4].

Geotextiles are made from polypropylene (PP), polyester (PET), polyethylene (PE), high-density polyethylene (HDPE), polyamide (nylon), polyvinyl chloride (PVC), and fiberglass; and PP and PET are the most widely used materials [5]. GSM, the metric measurement of the weight of a fabric (g m^{-2}), varies from under 40 to over 3000. The basic forms [4] of geotextile fabrics are: (i) woven, (ii) knitted, and (iii) nonwoven (fibers are bonded either by friction and/or cohesion and/or adhesion). There are variations of nonwoven structures: spun bonded, needle punched, thermal bonded, melt blown, etc.

Coastal erosion is a problem significant on the global scale . To protect or minimize damage from erosion, a geotextile compound is used in various structures: embankments, gabions, seawalls, offshore breakwaters, artificial reefs, and other. Geotextile is more effectively used for reinforcement in construction of embankments and retaining walls in soft soil and sand [4]. Geotextiles, being a relatively thin light weight material, when used in the marine and coastal environment is subjected to severe abrasion from armature rock and marine sediment, as well as from both wind action and wave impact. Consequently, geotextiles used in coastal and marine environment must be able to resist extremely harsh and adverse conditions [5].

As examples, some applications of geosynthetics as measures for coastal protection or coastal engineering could be mentioned: (i) geotextile tubes for the purpose of coastal defense appearing in the last decade in India [5]; (ii) geotextile containers at Adani Port, Gujarat, India ; (iii) Geotextile Sand Containers (GSCs /or Geobags) in coastal protection structures in Australia and Germany [6, 19]; (iv) coastal revetment (TenCate geotextiles underneath the riprap), East Lyme in Connecticut, USA [5]; (v) TenCate geotextiles for the fabrication of foundation mattresses, Eastern Scheldt Barrier, the Netherlands [5]; (vi) coastal protection at Lombang Beach, Indonesia [5]; (vii) revetment of Thorpeness beach, Suffolk, UK [5], etc.

The application of geosynthetics in hydraulic engineering leads to its increasing durability and environmental sustainability compared to alternative projects [6]. It is generally recognized that the treatment of geosynthetic materials with antioxidants significantly prolongs their service life, especially when used in conditions with low temperatures and with limited access to oxygen. But over time, geomaterials lose their properties (closely related to the aging of geosynthetic products), degrade and become a source of plastic contamination in the marine environment. In addition, plasticizers or antioxidants (additives to materials) can be emitted into the environment [7].

Apparently geotextiles degradation processes are similar to processes other plastics are subject to in the marine environment because they are based on the same types of polymers. Specific density of most plastics found in marine environment ranges between 0.05–1.70 g cm^{-3} [8, 9, 10] and the density of sand is around 2.65 g cm^{-3}. Plastic debris is exposed to environmental forcing and begins to degrade slowly [10, 11, 12, 13] generating a huge number of macro-, micro-, and nano-particles. These particles are freely transported by water flows and have adverse effects on the environment [10, 14].

978-1-5386-4467-6/18/$31.00 ©2018 IEEE

The issue of plastic debris in the ocean, emission of additives and their degradation products from the polymeric material into the environment has found considerable public attention [6]. With time, geosynthetic materials will become brittle due to aging, they will rupture and disaggregate. However, it is difficult to obtain reliable data [6].

In all publications the particular attention is paid to the structure, reliability, stability, and service life of engineering structures containing geosynthetic materials [6]. Special attention was paied to ecotoxicological effect on environment [7]. Literature review didn't give us any examples of studies on distribution of degraded geosynthetic materials from deformed or destroyed coastal protection structures.

The main purpose of the present study is to report on a new type of sandy beach contamination, namely, on geosynthetic pollution of the shore of the Sambia Peninsula (Kaliningrad Oblast of the Russian Federation, the Baltic Sea, Fig. 1).

II. Study Area

The Baltic Sea shore within Kaliningrad Oblast is 147 km long, composed of sandy beaches (the Curonian and the Vistula Spits, the western shore of the Sambia Peninsula from Baltiysk to Yantarnyi) and a complex of glacial and water-ice deposits (cliffs) on the northern shore of the Sambia Peninsula [15, 16]. The Baltiysk and the Klaipeda Straits connect the Vistula and the Curonian Lagoons with the Baltic Sea. The study area includes the shore of the Sambia Peninsula: from Yantarny to the root of the Curonian Spit (nearby Zelenogradsk) (Fig.1). The shores are influenced by severe storms in the autumn-winter period caused by northern and western winds [16, 17].

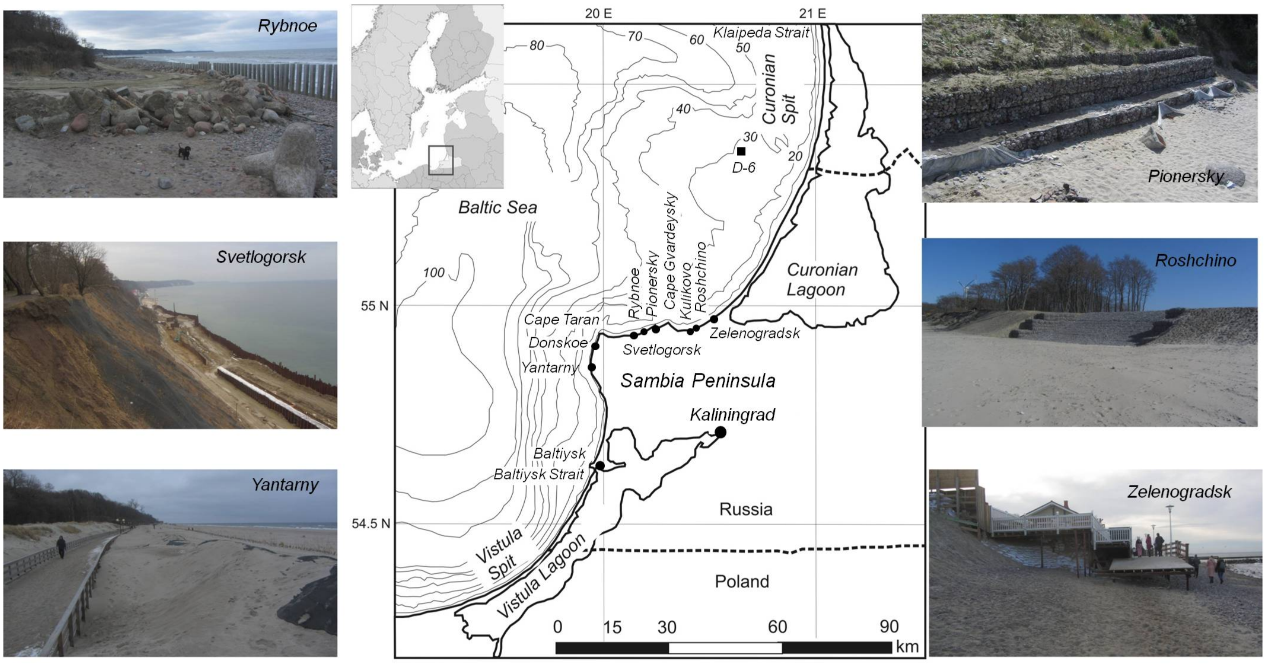

Fig. 1. Study area in the South-Eastern Baltic (Kaliningrad Oblast of the Russian Federation) and locations where protection constructions containing geotextiles exist (*Photo by Esiukova E.*).

III. Materials and methods

During the period from October 22, 2017 to March 31, 2018 the Baltic Sea shore of the Sambia Peninsula (Fig.1) was monitored to establish the contamination rate of sandy beaches by debris of geotextiles. The number of visits per each site was the following: Svetlogorsk (4 times), Pionersky (3), Zelenogradsk (4), Kulikovo (3), Roshchino (3), "Amber" gambling zone (4), Rybnoe (1), Cape Taran (2), Yantarny (3). These sites were the regular focus areas during the previous study on meso/macroplastic and paraffin pollution [18]. Most observations (October 2017 – March 2018) were made during or after storm events. Observations covered the entire part of the beach at a distance from 500 m to 3 km. The sampling of geotextile was accompanied by fixation of coordinates, photo documentation and description of the place of sampling. The size of the samples was from 25 mm to 1 m, but mostly the length of the samples was about 3–50 cm. In case the large pieces (more than 1-2 m), the fragments of textiles with visible different state of degradation were cut off from them. More than 40 samples of geotextiles were handpicked during 10 surveys. In 9 surveys, no debris of geotextiles was found, in 6 surveys the geotextile pieces were found in a

hard-to-reach place (in the water or on a slope). We may assume that small geotextile pieces were not seen in algae heaps or under a layer of sand and pebbles.

IV. SOURCES OF GEOTEXTILE

Several local sources of pollution of beaches by geosynthetic materials were identified, namely, the coastal protection constructions located on the shore of the Sambia Peninsula.

Yantarny: Knitted PP covers sandy embankments near the new boardwalk (far from the sea line). During the autumn-winter period of 2017–2018 with extremely powerful storms (for example, October 27–29, 2017 – Storm Herwart) the shore was strongly damaged, and the boardwalk was covered with a thick layer of sand. The sand was removed from the boardwalk and was covered and fixed by geotextile to avoid its further migration. The edges of black or dark gray knitted PP geotextile sheets were deformed in some places (Fig. 2).

Svetlogorsk: Geosynthetic materials (for examples, nonwoven geotextile from PP or PET fibers) are used here in coastal protection embankments, gabions and erosion control mats. Geomats (thick PP filaments, three-dimensional structure) are fixed on long and steep cliff slopes to prevent land sliding and facilitate more efficient vegetation (Fig. 3).

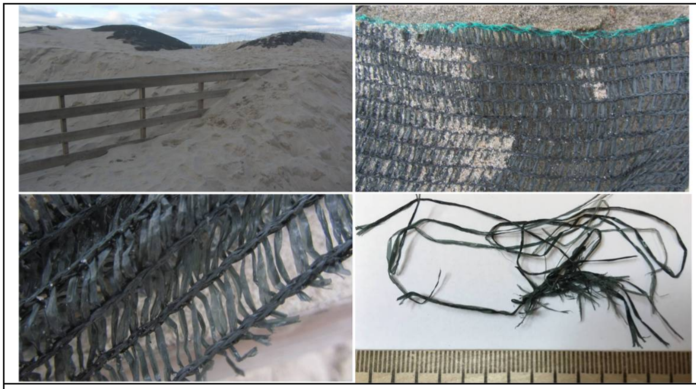

Fig. 2. Knitted geotextiles cover sandy embankments in Yantarny (*Photo by Esiukova E.*).

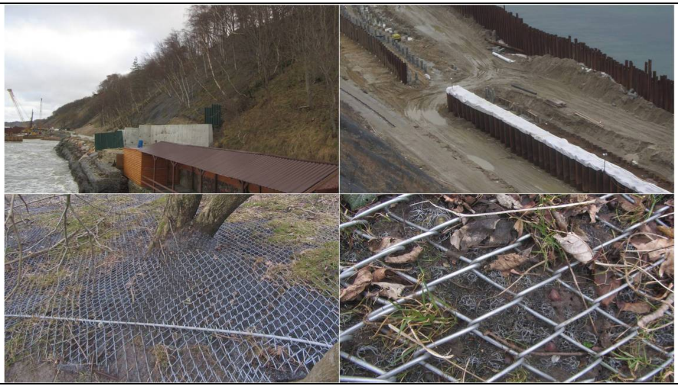

Fig. 3. Geomats cover slopes and are fixed by metal net on top as well as white nonwoven geotextile at the base of the promenade in Svetlogorsk (*Photo by Esiukova E.*).

Rybnoe: White and beige nonwoven geotextile (from PP/PET fibers) is at the base of the embankments of the promenade, which was built in the western part of the village of Rybnoe (Fig. 4).

Pionersky: A slope next to the promenade of the “Yantar” Residence was reinforced with gray non-perforated flexible geocells made from PP, HDPE or PE fibers. This slope was partially destroyed during the extremely powerful storm on October 27–29, 2017 (Storm Herwart). Flexible geocells were destroyed and transported along the beaches under the influence of waves, wind and currents (Fig. 5a). In several places along the shore of Pionersky the gabions which contain non-woven white geotextiles (PP / PET fibers) were built to protect slopes (Fig. 5b-d).

Zelenogradsk: In the western part of the city of Zelenogradsk, at the end of promenade, stacks of white woven sandbags (probably, from PP or HDPE fibers) were used to prevent erosion of the basements of cafes and restaurants built on the beach (Fig. 6).

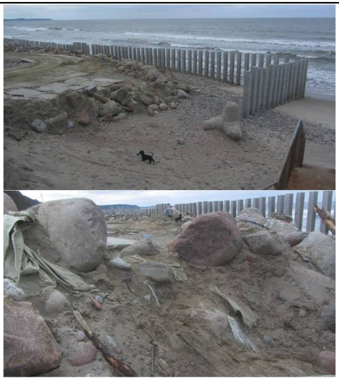

Fig. 4. White and beige nonwoven geotextile at the base of the promenade in Rybnoe (*Photo by Esiukova E.*).

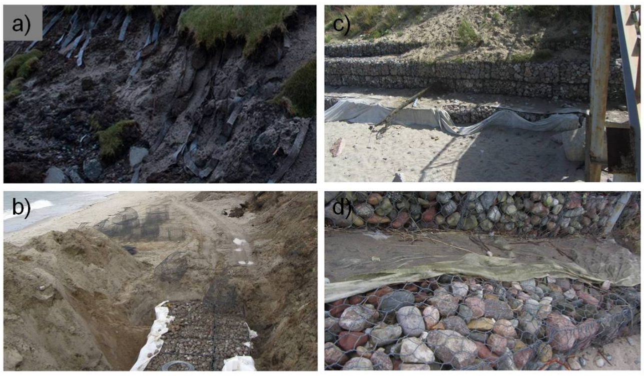

Fig. 5. Gabions in Pionersky: destroyed geocells on the slope (a) and white nonwoven geotextile at the base of gabions (d-c) (*Photos by Esiukova E.*).

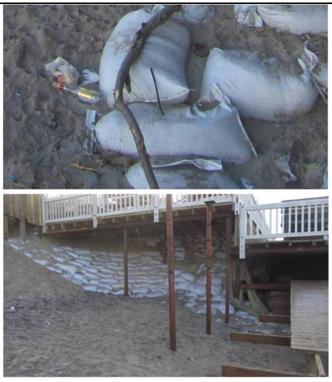

Fig. 6. White woven sandbags in Zelenogradsk in the basement of beach buildings (*Photo by Esiukova E.*).

The monitoring of the shores showed that most structures contain shabby geosynthetic materials. For example, the gabion construction in the east part of Pionersky was built in February 2016 (Fig. 1, Fig. 5b), and two years later the nonwoven geotextile used in the basement and around the boulders layers was completely deformed, torn off, and it turned green (Fig. 5c,d).

Many builders of the coastal protection structures use FIBC (Flexible Intermediate Bulk Container) bags, the soft containers with ribbon slings (PP, HDPE, PA) with carrying capacity from 300 kg to 3000 kg, for delivery of goods to the coastal zone (including cement, sand, dry mixes). Woven bags with sand-gravel mixture are used for erosion control in the coastal zone. These deformed and torn bags get into the environment under the influence of external conditions and storm episodes (Fig. 7a-c).

Only the slope protection in Svetlogorsk is still in good shape. A lasting covering material reinforced with steel mesh on top is partially covered with grass.

Apparently, some other embankments, retaining walls, gabions, waveguide bands and other structures may contain geotextiles, but the initial examination did not reveal this. Probably, the visible absence of geotextiles in the bases of gabions, barrage walls, waveguide bands, and stone breakwaters is associated with the buried position of materials into the beach or under water.

V. Results and discussion

During the period from October 2017 to March 2018 more than 150 samples of geosynthetic materials were collected (excluding fragments of big bags). Dornit geotextile makes up about 47% of all collected geosynthetics samples, plastic coating from gabions – about 27%, fragments of geo cells (geotextiles) – about 13%, geocomposites (geomats) – about 5%, other types – 1–3%. It turned out that the distribution of geosynthetic debris along the surveyed areas of the coast is irregular.

Large pieces of geotextiles (mega/macro-sizes, i.e. greater than 1 m / 2.5 cm, accordingly) and smaller pieces (meso/micro-size, i.e. greater than 5 mm / 0.5 mm, accordingly), being the debris of geotextile from the hydraulic structures, are transported by alongshore currents. For example, fragments of geocells that were torn from the slope in Pionersky during the storm on October 27–29, 2017 were detected east of Pionersky as far as 7 km away (near the village of Kulikovo) one month later, and 3.5 months later – as far as 9 km (near the village of Roshchino). For five months, they were present on the beaches from Pionersky to Cape Gvardeysky (Fig. 7d,e), a 3-km section of the shore, some of the geocells (mostly large sections of cells in a relatively good shape) were found at the base of the cliffs and in the middle of the beach on its surface (Fig. 7f,g). Most of the shabby scraps of geocells were located in the wracklines, in the swash zone (Fig. 7h,i), in heaps of other anthropogenic debris, mostly scraps of woven bags.

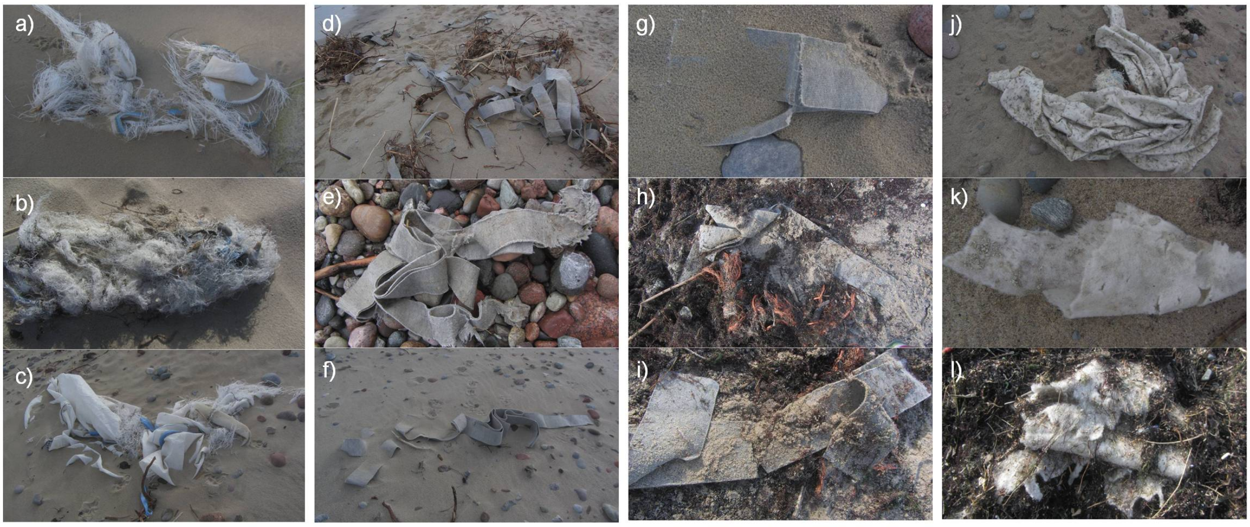

Fig. 7. Deformed geotextiles (from coast protection structures) which migrated along the shore: woven bags (a-c), geocells from the slope in Pionersky (d-i), nonwoven geotextiles (j-l) (*Photos by Esiukova E.*).

The pieces of nonwoven geotextiles (PP, PET) from gabions (Fig. 7 j-l) in Pionersky and Svetlogorsk were found very often buried below a layer of sand and soil. For example, severely torn, dirty, partially greenish pieces of white polypropylene geotextile (looking like felt) were scattered in large numbers in the vicinity of the villages of Rybnoe and Kulikovo, along the shore segment from Svetlogorsk to the village of Roshchino.

Intense contamination with remains of woven bags (HDPE, PP) (Fig. 7 a-c) was discovered at the shore segment from the village of Kulikovo to Zelenogradsk, as well as at some points at the root of the Curonian Spit.

It is noted that eolian transport, migration of sand along and across the beach leads to the complete or partial burial of geosynthetic material under the sand. This indicates that we sometimes just can't notice the presence of such debris on the beach, although it remains in the beach body until the next destructive storm episodes. On the surface of the beach geotextiles are exposed to abrasion and mechanical deformation, UV-induced photodegradation reactions, thermal reactions, hydrolysis, as well as microbial biodegradation / biofouling.

For now we have not found any data in the literature for an initial comparative analysis. The accepted basic methods for assessing the pollution of beaches with various types of anthropogenic debris and plastics (for example, OSPAR [20], etc.) do not allocate geotextiles or geosynthetics to any independent category, they are categorized as "other textiles" or "plastic fragments".

This specific pollutant could be attributed to damaged coastal protection constructions, and the length of polluted shore may be tens of kilometers. Traditionally geotextile is used in waste containment facilities [21]. Its usage in the coastal protection constructions leads to threat of pollution of shores by debris of geotextile as these constructions are placed in conditions of hard dynamic impact and can be destroyed by wave and currents with high probability.

IV. Conclusions

The example of the Sambia Peninsula (Kaliningrad Oblast, Russia) demonstrated that the wide use of geotextile materials in the coastal protection constructions may result in a new type of beach pollution. Surveys of the shore of the Sambia Peninsula (Kaliningrad Oblast, Russia) in the late autumn and winter stormy period (November 2017 – March 2018) showed that debris of geosynthetic materials used in coastal hydraulic structures were found along the 50 kilometers shore segment (from the settlement of Yantarny to the root of the Curonian Spit).

Analysis of distribution of geotextile debris along the shore allowed us to make a few conclusions of local importance. The attribution of different samples of geotextile debris to their possible sources gave the following: black cover PP material of knitted geotextile came from constructions in Yantarny, white PP/PET fiber geomaterial came from the bases of the promenade in Svetlogorsk, white PP or HDPE sandbags were transported from the basements of cafes in Zelenogradsk. The most worn out and deformed geotextile pieces came from the bases of gabions in Pionersky and Svetlogorsk, from the base of the embankments in the village of Rybnoe. The use of geotextiles in the construction next to the village of Roshchino, the presence of geosynthetics in the gabions next to the Cape Gvardeysky, in the base of coast protection structures near Zelenogradsk, as well as the retaining wall in the area of sewage treatment plants in the settlement of Yantarny still require clarification.

It is noted that worn geotextile is broken under the wind-wave action, and its parts migrate over long distances along the shore of the region. This geotextile undergoes photodegradation, thermooxidation, hydrolysis, biodegradation, abrasion, and thus becomes torn and breaks up into smaller parts. Fibers from disheveled geotextiles are easily separated and transferred into another size range – from mega/macroplastics to meso/microplastics. Therefore, it is necessary to remove all deformed geotextiles completely from the beaches to prevent their further decay and clogging of the coastal areas as geotextile containing plasticizers and stabilizers might contaminate the environment.

The next step of the study will be the survey of coastal engineering structures and the quality of geotextiles from hydraulic structures and beaches in order to establish the degree of safety of these materials for the environment and human beings.

Acknowledgement

The investigations of geotextile debris were supported by RFBR 18-55-76002 ERA_a grant within the ERANET-Rus joint project EI-Geo.

References

[1] *Hand Book of Geotextiles* (Special Publication 8.2.34). Bombay Textile Research Association (BTRA): Bombay, 187 p, 2012.

[2] M.E.H. Nizam, S.C. Das, "Geo Textile - A Tremendous Invention of Geo Technical Engineering," IJASGE, vol. 3(3), pp. 221-227, 2014.

[3] M.J. Denton, P.N. Daniels, *Textile Terms and Definitions*, 11th Ed., The Textile Institute, Manchester. 2002.

[4] C.H. Das, D. Paul, M.M. Fahad, T. Islam, M.E.H. Nizam, "Geotextiles – A Potential Technical Textile Product," *J. sci. eng. res.*, vol. 4(10), pp. 337-350, 2017.

[5] A. Mitra, "Application of geotextiles in Coastal Protection and Coastal Engineering Works: An overview," *Int. Res. J. Environment Sci.*, vol. 4(4), pp. 96-103, 2015.

[6] W.W.Müller, F. Saathoff, "Geosynthetics in geoenvironmental engineering," *Sci. Technol. Adv. Mater*. vol. 16 (3), pp. 034605, 2015.

[7] B.V.Wiewel, M. Lamoree, "Geotextile composition, application and ecotoxicology—A review," *J Hazard Mater*. vol. 317, pp. 640-655, 2016.

[8] I. Chubarenko, A. Bagaev, M. Zobkov, E. Esiukova, "On some physical and dynamical properties of microplastic particles in marine environment," *Mar. Pollut. Bull.*, vol. 108, pp. 105-112, 2016.

[9] V. Hidalgo-Ruz, L. Gutow, R.C. Thompson, M. Thiel, "Microplastics in the marine environment: A review of the methods used for identification and quantification," *Environmental Science and Technology*, vol. 46, pp.3060-3075, 2012.

[10] A.L. Andrady, "Microplastics in the marine environment," *Mar. Pollut. Bull.*, vol. 62(8). pp. 1596-1605, 2011.

[11] J. Wang, Z. Tan, J. Peng, Q. Qiu, M. Li, "The behaviors of microplastics in the marine environment," *Mar. Environ. Res.*, vol. 113, pp. 7-17, 2016.

[12] K.L. Law, "Plastics in the Marine Environment," *Annu. Rev. Mar. Sci.*, vol. 9, pp. 205-229, 2017.

[13] M. Tosin, M. Weber, M. Siotto, C. Lott, F. Degli Innocenti, "Laboratory test methods to determine the degradation of plastics in marine environmental conditions," *Front Microbiol.*, vol. 3, pp. 1-9, 2012.

[14] P.J. Kershaw (Ed.), *Sources, fate and effects of microplastics in the marine environment: a global assessment*. Rep. Stud. GESAMP 90, 96 p, 2015.

[15] T.A. Łabuz, "Environmental Impacts - Coastal Erosion and Coastline Changes," In: The BACC II Author Team (Eds.), *Second Assessment of Climate Change for the Baltic Sea Basin, Regional Climate Studies*. Springer, Berlin, pp. 381-396, 2015.
[16] J. Harff, S. Björck, P. Hoth (Eds.), The Baltic Sea Basin. Springer, Berlin, 2011.
[17] V.P. Bobykina, Zh.I. Stont, "Winter storm activity in 2011–2012 and its consequences for the southeastern Baltic coast," *Water Resources*, vol. 42, pp. 371-377, 2015.
[18] E. Esiukova, "Plastic pollution on the Baltic beaches of the Kaliningrad region, Russia," *Mar. Pollut. Bull*., vol. 114, pp. 1072-1080, 2017.
[19] J. Saathoff, H. Oumeraci, S. Restall, "Australian and German experiences on the use of geotextile containers," *Geotextiles and Geomembranes Journal*, vol. 25, Elsevier, pp. 251–263, 2007.
[20] OSPAR Commission. Guideline for Monitoring Marine Litter on the Beaches in the OSPAR Maritime Area, ISBN 90 3631 973 9, 2010.
[21] A. Bouazza, J. G. Zornberg, D. Adam. "Geosynthetics in waste containment facilities: recent advances". In: *Geosynthetics - 7 ICG.* Delmas, Gourc & Girard (eds) 2002. Swets & Zeitlinger, Lisse ISBN 90 5809 523 1.pp. 445-507.
